# Current Perspective on the Therapeutic Preset for Substance-Assisted Psychotherapy

**DOI:** 10.3389/fpsyg.2021.617224

**Published:** 2021-07-13

**Authors:** Sascha B. Thal, Stephen J. Bright, Jason M. Sharbanee, Tobias Wenge, Petra M. Skeffington

**Affiliations:** ^1^College of Science, Health, Engineering and Education, Murdoch University, Perth, WA, Australia; ^2^School of Medical and Health Sciences, Edith Cowan University, Perth, WA, Australia; ^3^Psychedelic Research in Science and Medicine Pty Ltd (PRISM), Balwyn North, VIC, Australia; ^4^Department of Psychology and Criminology, School of Arts and Humanities, Edith Cowan University, Joondalup, WA, Australia; ^5^International Society for Bonding Psychotherapy, Friedrichshafen, Germany

**Keywords:** substance-assisted psychotherapy, psychotherapy, psychedelics, therapeutic rationale, therapeutic conduct

## Abstract

The present narrative review is the first in a series of reviews about the appropriate conduct in substance-assisted psychotherapy (SAPT). It outlines a current perspective onpreconditions and theoretical knowledge that have been identified as valuable in the literaturefor appropriate therapeutic conduct in SAPT. In this context, considerations regarding ethics and the spiritual emphasis of the therapeutic approaches are discussed. Further, current methods, models, and concepts of psychological mechanism of action and therapeutic effects of SAPT are summarized, and similarities between models, approaches, and potential mediators for therapeutic effects are outlined. It is argued that a critical assessment of the literature might indicate that the therapeutic effect of SAPT may be mediated by intra- and interpersonal variables within the therapeutic context rather than specific therapeutic models *per se*. The review provides a basis for the development and adaptation of future investigations, therapeutic models, training programs for therapists, and those interested in the therapeutic potential of SAPT. Limitations and future directions for research are discussed.

## Introduction

Research on psychotherapy utilizing psychoactive substances, subsequentially referred to as substance-assisted psychotherapy (SAPT), has been re-established in the 21st century. This renewed interest has been referred to as the “psychedelic renaissance” (Sessa, 2012). It has followed a period in which these substances were stigmatized, not due to their international prohibition in 1971 since the early evidence collected on SAPT suggested psychedelics could be effective in treating a variety of mental health conditions; but rather, due to the circumstances in which they were prohibited (Grinspoon and Bakalar, 1979). With public and professional interest in SAPT rapidly growing, this narrative review presents the perspective on the necessary preset (i.e., valuable preconditions and knowledge) for practicing SAPT that has been identified in the current literature on the topic.

The recent growth in research has included clinical trials demonstrating the safety and efficacy of this approach for treatment refractory post-traumatic stress disorder (PTSD; Mithoefer et al., 2019), alcohol dependence (Bogenschutz et al., 2015), as well as anxiety and depression associated with end stage cancer (Agin-Liebes et al., 2020). Open label trials have also indicated that psychedelic-assisted psychotherapy could be effective in treating obsessive–compulsive disorder (Moreno et al., 2006), treatment-resistant depression (TRD; Carhart-Harris et al., 2016), and nicotine dependence (Johnson et al., 2017). Although there is recent progress in recording the safety and effectiveness of these approaches paving the way for their potential implementation and application in clinical practice (e.g., Kargbo, 2020; Nichols, 2020), the mechanisms responsible for therapeutic change across different psychotherapeutic approaches are still not fully understood.

Classic psychedelics (e.g., LSD, psilocybin, DMT and mescaline) are regarded as non-addictive and do not appear to present an independent risk factor for mental health problems (Krebs and Johansen, 2013; Hendricks et al., 2015; Johansen and Krebs, 2015). Meanwhile, other psychoactive substances with subjective effects that are similar to psychedelics, such as ketamine (Krupitsky and Grinenko, 1997; Serafini et al., 2014), MDMA (Thal and Lommen, 2018) and ibogaine (Alper, 2001), are being investigated as for their potential use in SAPT.

Classic psychedelics and MDMA have been found to produce acute and persistent positive personality changes (Bouso et al., 2018) and improved well-being in healthy individuals (Elsey, 2017; Jungaberle et al., 2018). These substances have been linked to heightened mindfulness (Soler et al., 2016, 2018), a sense of connection to nature (Forstmann and Sagioglou, 2017), creativity (Kuypers et al., 2016; Uthaug et al., 2018; Mason et al., 2019), and openness (Liechti et al., 2017). Many researchers have called for more extensive research into the potential of the clinical applications of these substances, particularly in respect to the current mental health crisis (see Belouin and Henningfield, 2018; Schenberg, 2018) and the limited awareness of their potential amongst health care professionals (Barnett et al., 2018).

It is becoming increasingly apparent that the potential effects of SAPTs are dependent on several contextual factors, including psychological preparation prior to the experience (Johnson et al., 2008), extra-pharmacological manipulations in the form of specific music playlists (Kaelen et al., 2018), and psychological integration subsequent to the experience (Hartogsohn, 2017; Richards, 2017; Carhart-Harris et al., 2018; Garcia-Romeu and Richards, 2018). Key components that meditate the therapeutic alliance (e.g., unconditional acceptance and therapeutic presence) and have been shown to be strong predictors of outcome in traditional psychotherapy (Frank and Frank, 1991; Geller and Greenberg, 2012; Norcross and Lambert, 2018), are also likely play a vital role in SAPT (Greer and Tolbert, 1998; Garcia-Romeu and Richards, 2018).

In this narrative review, we will summarize valuable considerations, methods, models, and current (theoretical) concepts regarding the psychological mechanism of action and therapeutic effects of SAPT that are identified in the literature. Thereby, we highlight similarities between models and approaches to outline potential mediators for therapeutic effects. Further, we critically discuss literature indicating that various schools of psychotherapy may translate and incorporate their approaches to SAPT. This article is intended to provide the preface (or preset) to a series of systematic and narrative reviews examining each stage of the SAPT treatment process, by outling the valuable prerequise knowledge for psychotherapists interested in practicing SAPT. In doing so, we have not considered the additional medical training and knowledge required to provide SAPT (e.g., physiological conditions and concomitant mediucations that would contraindicate SAPT) in this article since it is not within our scope of clinical practice.

## Ethical Considerations

There are strict conditions and frameworks required by ethical committees of universities and research institutes currently investigating SAPT, extensive bureaucratic and jurisdictional exchange with government institutions, and cautious storage, documentation, and safety procedures are necessary to conduct research in this field (Strassman, 2001; Johnson et al., 2008; Bogenschutz, 2013; Nutt et al., 2013). While standard ethical guidelines apply to SAPT, additional considerations are vital. The Council on Spiritual Practices offers a code of ethics for spiritual guides that provides a framework for guiding people through exceptional states of consciousness that can be transferred to therapeutic sessions with psychoactive substances (Jesse, 2001). Therein, it is firmly highlighted that it is crucial for therapists to be well-intended and empathetic towards their clients due to augmented transference, counter-transference, and suggestibility (Leary, 1961; Fadiman, 2011; Carhart-Harris et al., 2015). As such, risks (both physical and psychological) should be discussed, preventive measures should be taken, and the client should be educated on the comparatively rapid transformative potential of the therapy (see Griffiths et al., 2006).

Therapists should be aware of the possibility of substance-occasioned uncomfortable material (sexual, or traumatic), and be prepared to respond to this using methods and techniques for which they are qualified through education, training, and experience (Grof, 2000; Phelps, 2017). In these situations, therapists need to simultaneously help clients process this material and prevent colluding with clients in acting out inappropriately (see Grof, 2000). Therapists and clients should agree upon limits on behaviors prior to the session. Such limitations could include outlining the parameters for appropriate touch, prohibiting self-harm, violence against facilitators and others, destruction of property, and sexual acting out (Mithoefer, 2017).

Increased vulnerability to suggestions and manipulations due to the heightened suggestibility in psychedelic states was identified in healthy volunteers after the administration of LSD (Carhart-Harris et al., 2015). This vulnerability, combined with increased attribution of certain ideas holding personal meaing or perceiving the ideas and contents experienced under the influence of psychedelics to hold greater noetic truth value in both healthy and clinical samples (Timmermann et al., 2020), may lead to reduced agency and judgment when compared to regular psychotherapy sessions. Even simple misunderstandings might be more likely to occur and the validation and mediation of the content and knowledge experienced in these states raises important ethical considerations: the increased risk of iatrogenic complications (e.g., false memory syndrome; Pope, 1996) through induced noetic feelings of revelation. Timmermann et al. (2020) propose a pragmatic framework founded on an ethical approach embedding these experiences within larger cultural- and historical contexts while considering their intersubjective character. They stress the importance of validation based on empathy by an experienced therapist to facilitate appropriate processing and integration of these experiences while considering aforementioned challenges. There is also an increased importance for therapists to be aware of the limitations on scope of practice required by most psychotherapy regulatory bodies, including preventing clients from being exploited, and therapists having an awareness of their own limitations, values, needs, and beliefs. Most importantly, therapists should be open for regular peer-review and seek council and feedback from other practitioners as required by most professional bodies. This is of utmost importance in SAPT because of the strict legal framework that must be considered and the aforementioned elevated potential for misperception and misjudgment.

The need for therapists’ self-experience with altered states of consciousness and substances that are administered in SAPT is controversial. For example, Forstmann and Sagioglou (2021) found that the perception of a reserachers’ integrity and quality of their research by psychedelic naïve participants was reduced if the researcher self-admitted substance use (Forstmann and Sagioglou, 2021). While there will likely be a continuum amongst therapists engaged in SAPT clinical trails, ranging from those without any personal experiences to those with a vast amount of experience across substances and settings, open discussion of these experiences can be constrained for fear of personal, professional, and sometimes legal consequences (Nielson and Guss, 2018; Ross et al., 2020). Some practitioners in the field regard personal experiences with these substances as essential in order to emphasize with the client’s experiences (Metzner, 1998; Bogenschutz, 2013; Bogenschutz and Forcehimes, 2017) and in certain countries, such as Switzerland, personal experience is required for therapists to work with clients in SAPT settings (Grof, 2000; Strassman, 2001). However, personal experiences may lead some therapists to become less curious about the client if they believe that their clients’ experience will be similar to their own (Bogenschutz, 2013; Bogenschutz and Forcehimes, 2017). Thus, personal experience is a double edged sword that may be a potential confound to research and SAPT, though has not yet been empirically investigated (Nielson and Guss, 2018). Also, in line with standard ethical guidelines, we believe that therapists should not take any mind-altering substances before or during therapy sessions, regardless of whether they are providing SAPT or regular psychotherapy.

## Spiritual Emphasis of the Therapy

Substance-occasioned spiritual or mystical experiences have been described as ineffable, noetic, and sacred, involving time dilation and ego dissolution (Griffiths et al., 2006, 2008; MacLean et al., 2011). They may be compared to non-substance-induced personal encounters with God, a divine entity, or Ultimate Reality (Griffiths et al., 2019). Such research using psychometric outcome measures was first conducted at Harvard overy 50 years using a 43-item tool develop by Pahnke (1969). More recent research has typically used a 30 item iteration of this tool (the revised Mystical Experience Qusetion; MacLean et al., 2012), which appears to have more robust psychometric properties (Barrett et al., 2015).

Since this therapeutic experience may be regarded as one of the most meaningful experiences of the clients’ lives (Griffiths et al., 2006, 2008; MacLean et al., 2011; Preller et al., 2017; Schmid and Liechti, 2018), Garcia-Romeu and Richards (2018) have proposed that therapists consider treating it accordingly throughout the entire therapeutic process. Adamson and Metzner (1988) have claimed that even though therapists may not be religious themselves and exclude these aspects from their usual work, spiritual perspectives on the experience that may arise in the clients engaged in SAPT should be respected and supported. While an emphasis on specific religious systems has been discouraged by Grof (2000), he has proclaimed it as useful to focus on the awareness of the aesthetic aspects of the world, basic philosophical questions of life, and the recognition of spiritual dimensions of existence in a non-specific way. Meanwhile, Stolaroff (2004) has propsoed that unless clients are using the word “spiritual,” it might be advisable to avoid labels, due to the danger of enhanced suggestibility. Importantly, avoiding the psychological blockages and focusing on the repetition of spiritual experiences (i.e., spiritual bypassing), may constitute a pitfall for therapeutic progress, with Frecska et al. (2016) cauitioning that it may result in avoidance of the real psychological issues and an unhealthy relationship with spiritual aspects of substance-induced experiences.

As outlined below, therapeutic approaches that integrate contemplative spiritual practices like third wave behavior therapies (TWBTs) have been identified as valuable frameworks for SAPT in the literature (Walsh and Thiessen, 2018). Recent research investigating the interaction between meditation and psilocybin found potential synergistic effects of meditation practices and mystical experiences or ego dissolution occasioned by psilocybin (Griffiths et al., 2018; Smigielski et al., 2019). This finding may suggest that the development of mindfulness- or other spiritual or meditative practices through the preparatory and integration stages of therapy could potentiate the treatment effects (Walsh and Thiessen, 2018).

## Mechanism of Action and Therapeutic Effects of Substance-Assisted Psychotherapy

It is predominantly believed that the therapeutic effects of substance-assisted treatments are a combination of the psychopharmacological effects of the substance (e.g., see Carhart-Harris et al., 2014; Carhart-Harris and Friston, 2019; Preller et al., 2019), which influence the client’s intrapsychic experience, but also the shared interpersonal experience between client and therapist (Adamson and Metzner, 1988), the mindset of the client and the therapist, and successful psychotherapeutic integration of the experience (Mithoefer, 2017; Bogenschutz et al., 2018; Malone et al., 2018). Thereby, the quality, intensity, and duration of challenging parts of the acute psychedelic experience may be key mediators for the therapeutic outcome (Carbonaro et al., 2016; Roseman et al., 2018). However, the acute and long-term or persistent effects (more relevant for treatment effects) differ substantially (Carhart-Harris et al., 2016), so the specific psychological mechanisms that account for their therapeutic efficacy are still not comprehensively understood. Various acute and persistent effects of psychedelics and entactogens were outlined by Jungaberle et al. (2018) and include, amongst others, positive effects on well-being, mood, empathy, cognitive flexibility, self-transcendence, and openness. However, individual experiences might be extremely variable and generalizations based on single sessions and small sample sizes are problematic (Grof, 2000; Bogenschutz et al., 2018) due to different personality structures of the client (Sandison and Whitelaw, 1957; Cohen, 1960; Gucker, 1963) amongst other individual differences. Consequently, attempts to depict and generalize the psychological mechanisms of action rendering SAPT an effective treatment option for certain disorders draw on similar terminologies to frame distinct theoretical concepts. These concepts may not be mutually exclusive (i.e., they tend to partially intersect). Thus, significant differences seem to be more evident in the theoretical foundations the concepts stem from rather than their conclusions. In this section, we have attempted to merge these concepts as derived from diverse models into meaningful clusters. We have also not attempted to distinguish the moderating factor of which psychedelics could differentially contribute to each mechanism, as the literature at present does not seem advanced enough for such an analysis.

### Personal Significance, Insights, and Meaningfulness

Early psychedelic research assumed that a heightened sense of significance and meaningfulness (also described as prolonged “eureka” experience) under the influence of certain substances contributed to the therapeutic effects (Ludwig and Levine, 1965b). Material encountered during substance-assisted sessions is usually personal and uniquely meaningful (Griffiths et al., 2006, 2008; MacLean et al., 2011; Preller et al., 2017; Schmid and Liechti, 2018) and relevant to the facilitation of personal growth in each client (Cutner, 1959; Shanon, 2003; Bogenschutz et al., 2018).

The importance of personal meaning has been highlighted in various empirical studies. In a randomized, double-blind, active placebo-controlled study, 29 participants experiencing cancer-related anxiety and depression received a single-dose of psilocybin in conjunction with psychotherapy (Ross et al., 2016). Through synthesizing prior qualitative analyses of interviews with these participants (Belser et al., 2017; Swift et al., 2017), with a qualitative analysis of their written narratives and their therapists’ notes, Malone et al. (2018) found that psilocybin may support clients in gaining insight regarding the root cause of their issues or disorders due to the personalized nature of the subjective experiences that seems to be tailored to individual psychological needs.

This hypothesis is supported from the results of a pilot study of 13 participants (*N* = 9 with diagnoses of substance abuse and/or dependence) in which self-report measures of the Purpose of Life Test significantly increased at three and nine-month follow ups after treatment with Ayahuasca (Fernández et al., 2013). In another study, 36 participants without prior experience with psychedelics received psilocybin or placebo in a double-blind counterbalanced design (Griffiths et al., 2006). In a 14-month follow-up, 58% of the participants assessed the experience as one of the five most personally meaningful experiences of their lives with 64% indicating persistent increases in life-satisfaction or well-being (Griffiths et al., 2008). Similarly, 13 of 15 (i.e., 86%) participants in a one-year follow-up of a psilocybin-facilitated smoking cessation trial rated the experience amongst the five most meaningful experiences of their lives (Johnson et al., 2017) and 10 of 14 (i.e., 71%) healthy volunteers in an LSD study rated the experience as one of the ten most meaningful experiences of their lives at one-year follow-up (Schmid and Liechti, 2018). Personally meaningful and emotional experiences with mystical qualities or ego dissolution are now thought to have causal links to persisting positive effects (Bogenschutz and Ross, 2016).

### Awe and Peak-Experiences

Awe can be defined as an emotion that is associated with profound pleasure bordering on fear while appreciating vastness (perceiving various stimuli as larger than the self) and accommodation (the need to revise mental structures to integrate novel experiences and perspectives; Keltner and Haidt, 2003). It is hypothesized that the profound awe that characterizes peak experiences occasioned by psychedelics may require habituated mental structures to accommodate to integrate and account for these novel perceptions and experiences. This process of cognitive re-accommodation may result in disorientation, fear, ego dissolution, and subsequent feelings of ultimate knowledge and enlightenment – once new information can be assimilated through the adaptation of mental structures (Hendricks, 2018). As a result, the profound feeling of awe may encourage one to superordinate the social collective to one’s individual needs, i.e., decrease egocentrism (Bai et al., 2017), and thus facilitate cooperation (Gowdy and Krall, 2015), shift attention to the presence while expanding experience of time (Rudd et al., 2012), and decrease tolerance for uncertainty which in turn elevates the inclination to acknowledge the influence of supernatural agents in experiences (Valdesolo and Graham, 2014). Thus, the sense of heightened connectedness or oneness with others and the universe (Carhart-Harris et al., 2018), and the shift of attention towards the collective and away from the self are theorized to constitute the positive treatment effects of psychedelics across a range of disorders (see Hendricks, 2018).

### Reconditioning

One potential mechanism of change in SAPT is the breaking of previously rigid maladaptive emotional and cognitive patterns, prior to them being reformed and reintegrated in a new structure (Frecska, 2011). This has been referred to as “restructuring,” “retraining” or “reconditioning” treatment (Spencer, 1964). The process can be seen as a “secure form of regression” (Frecska, 2011), in which old associations are broken down and unlearned. Thus, previously frightening stimuli could lose their association with threat, and become *deconditioned*, before being reintegrated in a less problematic way. This has also been described as “de-patterning” (Gasser et al., 2015). Neuroscientific evidence suggests that hyperexcitation in layer V pyramidal neurons mediated by the 5-HT2A agonism of psychedelics may disrupt fixated and conditioned cognitions, psychological habits, and constraints on internal generative models (Swanson, 2018). Disinhibition of these neurons may reduce the rigidity of higher-level beliefs (in a hierarchical predictive processing model) making them more receptive for bottom-up prediction errors (Carhart-Harris and Friston, 2019). Thereby, prior fixed beliefs may be more easily challenged and restructured by novel information. This might be reflected by lasting changes in attitudes (i.e., increased openness and extraversion and decreased neuroticism; Erritzoe et al., 2018) and beliefs in individuals who used psychedelics (Nour et al., 2017; Jungaberle et al., 2018).

### Perspective-Shifts and Cognitive Flexibility

Another proposed mechanism of action is perspective-shifts or cognitive flexibility. Seemingly, this mechanism can be interpreted as a combination of a restructuring or shifting of pre-existing concepts through experiences that carry emotional and/or intellectual value (see Section "Awe and Peak-Experiences"). New perspectives and insights might be gained and more rapidly accepted because of a novel sense of emotional and intellectual appreciation occasioned by the substance (Ludwig and Levine, 1965a). Some authors suggest that processing of significant emotional or intellectual content, combined with loosened ego functions in an altered emotional state, may allow for a reduction of ruminations and egocentricity (Northoff, 2007; Pizzagalli, 2011) wherein novel perspectives may be gained (Leuner, 1967; Carhart-Harris et al., 2014) and problems may be appreciated for their true significance (Hofmann and Ott, 1980). The potential for a client to experience an alternative view of themselves has been referred to as “stripping away layers of onions” by some therapists (Cattell, 1957, p. 227). Gasser et al.'s (2015) mixed methods data from 10 participants who received LSD-assisted psychotherapy suggest that amongst other variables, the cognitive experience (i.e., altered cognitive associations allowing for novel perspectives on intra- and interpersonal problems and relationships) mediates positive outcomes.

In a phenomenological discussion of the Ayahuasca experience, Shanon (2003), reported shifts in the locus of consciousness, self-consciousness, and personal identity (i.e., positive self; see Griffiths et al., 2008) that may result in heightened cognitive flexibility and support the adaptation to different contexts (Franquesa et al., 2018). Subjective feelings of increased connection with oneself were linked to positive treatment effects of Ayahuasca for substance use in an observational study of 12 participants in a retreat combining 4 days of group counseling and two Ayahuasca sessions (Thomas et al., 2013).

Ayahuasca and psilocybin were both shown to have acute and prolonged effects on cognitive-thinking style (Kuypers et al., 2016; Uthaug et al., 2018; Mason et al., 2019) and positive treatment effects of psilocybin may results from shifts in self-representation (Smigielski et al., 2019). Neurocognitively, these shifts may be linked to enhanced cognitive flexibility (Gallimore, 2015) and altered neural connectivity (Tagliazucchi et al., 2016; Carhart-Harris et al., 2017; Preller et al., 2019). This may also be reflected in changes in personality measures like decreased neuroticism and increased conscientiousness and extraversion (i.e., openness) that have been observed in an open-label trial of 20 participants with moderate to severe TRD (Erritzoe et al., 2018).

Qualitative data (Watts et al., 2017) from aforementioned open-label study investigating psilocybin for TRD (Carhart-Harris et al., 2016) suggests that psychedelics combined with psychotherapy may increase psychological flexibility more than psychotherapy itself (Watts and Luoma, 2020). It is suggested that increased psychological flexibility may mediate therapeutic effects (Hayes et al., 2012) in Acceptance and Commitment Therapy (ACT). This has led some authors (e.g., Sloshower et al., 2020; Wolff et al., 2020) to suggest that ACT might exhibit synergistic therapeutic mechanisms with SAPT. However, these speculations have yet to be verified.

### Mindfulness and Acceptance

Mindfulness and acceptance are important aspects of TWBT that share conceptual links and presumptions with the mechanisms outlined above. Namely, Wolff et al. (2020) proposed that shifts from experiential avoidance to acceptance (which is closely related to mindfulness-practices and a mediator of therapeutic effects in TWBTs) may be facilitated by the relaxation or disinhibition of higher level avoidance-related beliefs (see Sections “Reconditioning” and “Perspective-Shifts and Cognitive Flexibility”), elicitation and intensification of private events (see Section “Personal Significance, Insights, and Meaningfulness”), and operant conditioning of acceptance. During substance-assisted experiences, avoiding aversive aspects of the experience may increase adverse responses, while acceptance of the experiences may result in more rewarding responses (this was termed “avoidance sensitivity”). This results in the operant conditioning of acceptance instead of avoidance. The conditioned openness and acceptance may allow for exposure and consideration of private events that would otherwise be avoided. As a result, previously held beliefs may be revised during the relaxation of avoidance-related beliefs since this state allows for strong experiential contradictions to negative expectancies, i.e., the experience of oceanic boundlessness, blissful ego dissolution, and long-term increases in well-being (Wolff et al., 2020).

Substance-induced dissociation from one’s learned roles and habits coupled with a conflict-free state of being may foster acceptance of oneself. This hypothesis is supported by an independent group (*N* = 20 participants) self-report study comparing four consecutive Ayahuasca sessions with an eight-week mindfulness-stress reduction (MBSR) course (Soler et al., 2018). Results indicated that Ayahuasca and MBSR led to comparable increases in acceptance on the Non-Judging Subscale of the Five Facet Mindfulness Questionnaire. Meanwhile, a thematic analysis of semi-structured interviews of 20 participants’ accounts of an open-label trial of psilocybin for TRD at 6 months follow-up found change processes from disconnection (from the self, fellow human beings, and the world) to connection and from emotional avoidance to acceptance (Watts et al., 2017).

The potential for more open exploration of psychodynamic connections has been noted in early psychedelic research (Hausner and Dolezal, 1963). Further, decentering – observing one’s feelings and thoughts in a detached manner (Kerr et al., 2011) – was positively associated with Ayahuasca use in a self-report study of 41 participants without prior experience with the substance and 81 experienced participants (Franquesa et al., 2018). In a randomized, double-blind, placebo-controlled study of 38 participants in a mindfulness retreat, a single psilocybin experience was linked to alterations in mood, social behavior, spirituality, and attitudes about life (Smigielski et al., 2019). Long-term increases in mindfulness following a single dose of psilocybin in ten psilocybin-naïve participants persisted for at least 3 months, even without explicit mindfulness training. This increase in mindfulness may represent a key mechanism of psilocybin therapy (Madsen et al., 2020). Perceptual and qualitative changes regarding intra- and interpersonal consciousness and perceived mindfulness capacities have also been reported by clients who have received SAPT. Bogenschutz et al. (2018) have proposed that these changes mediate positive treatment effects.

### Re-assessment of Memories

Re-experiencing and re-assessing memories under the influence of certain substances may be another mechanism of action underlying SAPT that evidences conceptual links to personal meaning and perspective-shifts. This overlap of mechanisms may be most evident in the current rationale of the treatment effects of MDMA-assisted psychotherapy. MDMA increases cortisol, prolactin, oxytocin, dopamine, norepinephrine, and serotonin which in turn increases empathy and self-confidence and reduced feelings of anxiety and depression. This allows clients to revisit memories and experiences while staying emotionally engaged without being overwhelmed by negative emotions which may facilitate memory reconsolidation (Mithoefer, 2017; Thal and Lommen, 2018).

Based on clinical experiences with clients who have undergone SAPT with serotonergic psychedelics, Sandison (1959) has suggested that early memories could be re-experienced in vivid detail. While these memories are likely a mixture of facts and fantasies that contain archetypical experiences, these experiences (Spencer, 1964) may allow for acceptance of more important emotional connotations (see Sections “Personal Significance, Insights, and Meaningfulness” and “Mindfulness and Acceptance”) and thus result in personal growth regardless of their factual truth. Similarly, positive effects of abreaction itself under the influence of certain substances may not be dependent on the factual truth of the traumatic incident, but rather linked to the release and re-assessment of trauma-related emotions (see also Mithoefer, 2017). Curiously, describing specific episodic content of the psychedelic experience is usually more difficult than illustrating its cognitive and emotional impact (Bogenschutz et al., 2018). However, the ineffability of the experience does not seem to impact its treatment effects or its perceived meaningfulness (Malone et al., 2018).

In contrast, a placebo-controlled crossover study with 20 healthy participants by Speth et al. (2016) found that the intensity of the LSD experience was positively correlated with reduced mental time travel to past events. The authors argue that mind-wandering to past events may be more specific but less frequent under LSD. Since ruminating about past events is linked to reduced happiness (Killingsworth and Gilbert, 2010), psychedelics may display similarities to mindfulness-based therapies and thus foster mental focus on the presence (Shapiro et al., 2006; Soler et al., 2016, 2018). Gasser et al. (2015) found that, besides the cognitive experience, the psychodynamic experience (i.e., emergence of unconscious material into consciousness and/or reliving of past experiences) and the associated abreactions and catharsis plus the peak-experiences (i.e., ego dissolution, transcendence of time and space, and the sense of having meaningful novel insights), mediated positive outcomes in LSD-assisted psychotherapy. Intense emotional experiences with a state of well-being, pure presence (i.e., existing in the here and now), and freedom from past concerns, guilt, anxiety, and depression combined with the absence of former tensions had the most significant effect on clients. Similar experiences of psychodynamic material, catharsis, connection, love, and self-compassion have been reported in clinical investigations with psilocybin (Bogenschutz et al., 2018).

Neuroscientific research suggests that reactivation of past memories, reconsolidation through incorporations of new emotional responses related to these memories, and reintegration through altered behavior in everyday situations may result in therapeutic change in non-substance assisted therapy (Lane et al., 2015). It is possible that this similar mechanism contributes to the change in SAPT as well.

### Dreamlike Qualities

Some psychoactive substances have been associated with experiences with dreamlike qualities (Jacobs, 1978; Fischman, 1983; Wilkins et al., 2011; Carhart-Harris and Nutt, 2014; Morris and Wallach, 2014; Kraehenmann, 2017; Kraehenmann et al., 2017; Rodger, 2018; Sanz and Tagliazucchi, 2018) and it has been suggested (e.g., Kraehenmann, 2017) that the positive effects of dreaming on well-being and social functioning may also be applicable for psychedelics. It is further hypothesized that altered self-awareness occasioned by some substances may result from perspective-shifts (from first-person to third-person perspective) as is often reported in dreams (Thompson, 2014; Sanz and Tagliazucchi, 2018). Kraehenmann (2017) proposed that fear extinction and formation of novel extinction memories through associative learning in contexts that are not associated with fear (Nielsen and Levin, 2007; Levin and Nielsen, 2009) and memory reconsolidation (Schiller et al., 2010; Rolls et al., 2013) may be related to similar cognitive processes in psychedelics and dreams. These concepts overlap with deconditioning, and re-assessment of memories.

## The Development of Models for Substance-Assisted Psychotherapy

In the literature, models of SAPT have been distinguished primarily according to the dose of the active substance (Mangini, 1998). Low dose SAPTs, or *psycholytic therapy* (Pahnke et al., 1971), and high dose SAPTs or *psychedelic therapy*, sometimes referred to as *psychedelic peak-therapy* (Sherwood et al., 1968; Pahnke et al., 1971; Grof et al., 1973), each attempt to facilitate different processes. In *psycholytic therapy*, low to medium doses of psychoactive substances are used to foster the gradual and progressive unfolding of various levels of the unconscious activating and accelerating the process of psychodynamic or psychoanalytic psychotherapy (Leuner, 1967; Grof, 2000; Bogenschutz, 2013; Majić et al., 2015). Active verbal psychotherapy is conducted while the client experiences the effect of the substance that is used to facilitate revival and abreaction of past experiences and emotional releases, loosen defense mechanisms, and deepen introspection and insight. This is mediated by the therapist’s interpretation of unconscious material (Eisner and Cohen, 1958; Sandison, 1959; Leuner, 1967; Bogenschutz, 2013). Repeated sessions of SAPT (sometimes up to 80) are embedded within a psychodynamic or psychoanalytic psychotherapy process (with significantly more substance-free than SAPT sessions) stretching over several months or years (Chandler and Hartman, 1960; Leuner, 1967). This extensive time-frame renders the comparison to other models and the isolation of the effects of SAPT-sessions extremely difficult. Psycholytic therapy was employed as preferred treatment model for patients with less severe disorders, such as psychosomatic-, mood-, neurotic-, or personality disorders and those with an intellectual interest in the therapeutic process (Grinspoon and Bakalar, 1979; Gasser, 1995; Grof, 2000). Conveniently, the gradual unfolding of various layers of the unconscious may allow for unique insights across a variety of domains. In doing so, clients might achieve an enhanced understanding of different mechanisms and areas of the mind through which alterations in behavior or cognition may be achieved (Grof, 2000). Disadvantages include that this can require a long duration of treatment, and lower doses may mean clients ignore or resist more challenging qualities of the experience. Over the course of therapy, the experience of transference may inevitably intensify, leading to a deterioration of symptoms and decompensation. Finally, the conventional psychodynamic framework of the approach may be challenging to apply to more extraordinary experiences that transcend interpersonal and verbalizable realms (Grof, 2000). While psycholytic therapy has influenced current investigations into therapeutic applications of psychoactive substances, it has not been employed as the main model in recent clinical trials.

*Psychedelic therapy* employs high doses of psychoactive substances that substantially exceed those administered under psycholytic frameworks (although there is a certain variability to “low” and “high” doses in reported studies and standardized “high” and “low” doses are not distinctly defined). It assumes that the therapeutic effect is produced by the facilitation of the experience of ego dissolution, also referred to as mystical- or peak experience (Griffiths et al., 2006), thereby transcending the psychodynamic levels (Grof, 2000). As such, substances are only used in one to three sessions (guided by the intended effect and the model of treatment being used), while preparation- and integration sessions without substances may or may not be incorporated (Grof, 2000; Johnson et al., 2008; Bogenschutz, 2013). While psychotherapy is used to maximize the probability of these experiences to occur (Sherwood et al., 1968; Pahnke et al., 1971; Grof, 2000; Bogenschutz, 2013), the approach is not based on classic psychological theories (Leuner, 1967; Majić et al., 2015). The therapist supports the client in surrendering to and accepting the experience, but is non-directive and does not guide the client towards or away from particular experiences (Pahnke et al., 1971; Rhead et al., 1977; Bogenschutz, 2013). While music is provided, and therapists observe the client quietly, verbal interventions are usually not applied during the peak-substance effects (Bogenschutz, 2013).

Recent research has used the psychedelic approach (Bogenschutz et al., 2018; Nielson and Guss, 2018) as treatment for conditions such as addiction disorders and depression (Johnson et al., 2014; Bogenschutz et al., 2015; Carhart-Harris et al., 2016; Griffiths et al., 2016). The advantage of the model is that, due to the high doses used, clients may be less likely be able to resist the effect of the substances and may be more likely to completely surrender to the experience. This can allow for much more substantial effects in a single session compared to psycholytic therapy, which in turn makes psychological and environmental factors, i.e., set and setting (see Leary, 1961; Hartogsohn, 2016) more important for positive therapeutic effects. Due to its non-directive nature, there is no imperative for confrontation of psychodynamic conflicts. Instead, a greater depth, intensity, and spontaneity of the session has been described (Grof, 2000). Disadvantages may include the risk of greater emotional upheaval due to the more profound effects of the substances and greater physiological risks because of the higher doses (depending on the substance, of course). The non-directive approach may require the therapist to surrender to and support the process rather than focus on techniques and methodologies. It is mentioned that this requires a high level of expertise and trust in the process and that it is currently not clear whether improvements and transformations experienced in psychedelic therapies represent temporary shifts, or if deep structural psychodynamic changes may be attained. Although the general expectations may be higher in clients receiving this approach, it is impossible to guarantee profound experiences in all clients, which may lead to disappointment. The mechanism of action of therapeutic change is still not well understood and the entire experience can be difficult to capture, quantify, and integrate (Grof, 2000).

There is considerable overlap between certain fundamental assumptions of psycholytic and psychedelic models, leading some authors to suggest the approaches might be blended in practice to form an hybrid model (Mechaneck et al., 1968; Metzner, 1998). Non-directive approaches may be used during intense parts of the session, while discussion, interpretive work and meaning making may start before the effects of the substance have ceased completely (Masters and Houston, 1966; Grof, 2000; Bogenschutz and Forcehimes, 2017; Nielson and Guss, 2018). In this case, the adaptability of the therapist based on the client’s process would be vital.

In current models of MDMA-assisted psychotherapy for PTSD, this mixed approach has been recommended by Mithoefer (2017). The substance-assisted sessions are predominantly client-led; clients are encouraged to lie back with eyeshades on, focus inwards, and be present with their traumatic memories. Therapists gently guide clients to consider particular aspects of their traumas, and if necessary a short general discussion of the content of the session follows once the effects of the substance begin to subside (Mithoefer, 2017; Sessa, 2017; Sessa et al., 2019). There are reported exceptions to this approach. In a study by Wagner et al. (2019) clients alternated between spending time focused inside and time focused outside without headphones and eyeshades spend in conversation with their partner or therapists. In theory, all models for SAPT may allow for an incorporation of a variety of different schools of psychotherapy.

## Similarity Between Primary Mechanisms of Change in Various Forms of Psychotherapy and Mechanisms of Change in SAPT

Some of the mechanisms presented in the SAPT literature may also overlap with putative mechanisms of change posited in the non-SAPT psychotherapy literature. In this section we will relate the SAPT mechanisms to four key putative mechanisms, each highlighted as a central change mechanism by one of the major psychotherapy traditions: insight, cognitive restructuring, mindfulness, and experiencing.

Even though there has been a temporal shift from Freudian techniques to humanistic orientations (Grof, 2000), most of the therapies in the first wave of psychedelic research applied historically dominant psychoanalytic models (Roseman et al., 2018). Additionally, there have been case studies including use of exposure therapy (Costello, 1964), arguments for the application of learning theory for behavioral re-programming (Grieco and Bloom, 1981), and comparisons between approaches used in indigenous cultures using Ayahuasca and transpersonal, depth psychological, and parapsychological approaches and interpretations (Andritzky, 1989).

In recent studies, humanistic therapies such logotherapy (Ross et al., 2016), as well as CBT models (Johnson et al., 2014; Wolff et al., 2020) and third wave CBT approaches like ACT (Hayes and Wilson, 1994; Carhart-Harris and Nutt, 2017; Watts et al., 2017; Sloshower et al., 2020), DBT (which has been described as “behavioral translation of Zen”; Nuys, 2007) and mindfulness-based cognitive therapy (MBCT; Walsh and Thiessen, 2018) or motivational enhancement therapy (MET; Bogenschutz et al., 2015; Sessa et al., 2019) are used or being proposed as therapeutic frameworks. These therapies each attempt to target similar mechanisms to those identified as potential mechanisms in SAPTs.

### Insight in Psychodynamic Therapies

Insight has been considered to be a central mechanism of change, emphasized primarily in the psychodynamic tradition. Originally, Freud postulated that symptoms developed from repressing unacceptable material. From this perspective, a central mechanism of change involved making the repressed unconscious material conscious, especially regarding the historical developmental origins (Freud, 1920). Since the relational turn, this focus has shifted slightly, to specifically highlight the importance of insight into internalized relational configurations or models of self and other that developed from early experiences with attachment figures (Greenberg and Mitchell, 1983).

This focus on change through insight into previously repressed material, and insight into the effect of early relationships in psychodynamic treatments closely links to the SAPT mechanisms we have identified as *re-experiencing of memories* and *personal significance, insights, and meaningfulness*. Psychodynamic treatments would primarily use genetic interpretations into the developmental origins of the difficulty, and transference interpretations into the replaying of the relational patterns, to both identify and make meaning out of this material. However, with SAPTs we could see the initial process of insight being facilitated by the evocative function of the substance. The traditional meaning-making process of psychodynamic treatment would be highly relevant integrating the evoked material during a subsequent integration stage.

### Cognitive Restructuring in Cognitive Therapies

The central putative mechanism associated with Cognitive Therapy is changing beliefs through reasoning, or *cognitive restructuring* (Ellis, 1962; Beck, 1976). This mechanism has primarily been targeted by disputation (Ellis, 1962) and Socratic dialogues (Beck, 1976), in which people are lead to examine their beliefs through open questioning and evaluating evidence. This mechanism overlaps with what we have identified here as *reconditioning* and *perspective-shifts and cognitive flexibility*. While clearly, the initial shifts in SAPT come from substance induced evocation, not through reasoning processes, but the overlap of the identified mechanisms suggests that they may shift a common mechanism. This also implies that standard cognitive techniques may be relevant to consolidate the *restructuring* and *perspective shifts* during the integration stage of SAPTs.

### Mindfulness in Third Wave Behavioral Therapies

There are theoretical affinities between TWBTs (Hayes et al., 1999; Segal et al., 2002) and psychedelic therapy, such as their common root in contemplative spiritual practices, and the development of mindfulness as a putative mechanism of action (Soler et al., 2016, 2018; Smigielski et al., 2019). In TWBT, the psychological state of mindfulness is developed primarily through the practice of mindfulness techniques, such as imagining thoughts on a leaf in a stream, and meditation. While achieved through a different method, the resulting state of mindfulness may be similar to the state observed after the ingestion of psychedelics (Soler et al., 2016). This synergy suggests that mindfulness, acceptance, and meditation practices, like those included in TWBTs could be used to sustain the state of mindfulness during the integration stage of SAPTs (Walsh and Thiessen, 2018; Sloshower et al., 2020; Wolff et al., 2020).

### Therapeutic Relationship Conditions and Experiencing in Humanistic-Experiential Therapies

Humanistic-experiential therapies, such as Client-centered (Rogers, 1966), Gestalt (Perls et al., 1951), and Emotion-Focused Therapy (Greenberg and Johnson, 1988; Greenberg et al., 1993) have emphasized two key change mechanisms – the therapeutic relationship conditions and client experiencing – as central to the psychotherapeutic change. Rogers (1959) posited that when someone perceives that others’ positive regard for them is conditional, then they develop “conditions of worth” reflecting how they need to be in order to be accepted. Symptoms then manifest from needing to distort experience to be congruent with these conditions of worth. From this perspective, healing and growth can occur when therapeutic relationship conditions, including empathy, unconditional positive regard, and congruence (Rogers, 1957), mean that a client does not need to defensively distort their experience to be accepted. Thus, it is the therapist providing the relational conditions, as the soil for a seed, that would allow a client to change.

The second change mechanism is *client experiencing*, which is that healing comes from accessing, feeling and symbolizing the previously distorted or disavowed emotions. In recent versions of experiential theory, this has been described as “changing emotion with emotion,” such that accessing previously disavowed emotions is what changes the problematic emotions. For example, shame could be changed by accessing assertive anger or compassion (Greenberg, 2015). This requires the safety provided by therapeutic conditions as a necessary prerequisite, but also then suggests that actively directing clients towards their emotional experience through internal focusing (Gendlin, 1996), or through the use of evocative imagery and enactment tasks, such as chairwork (Greenberg et al., 1993; Perls, 1996), further facilitates the change process.

These two mechanisms may also overlap with some of the identified SAPT mechanisms, as well as the technique suggestions for conducting SAPT. Firstly the perception and receptiveness to the Rogerian relational conditions could be facilitated by some substances, such as MDMA (Thal and Lommen, 2018). The *experiencing* mechanism also seems closely tied to the SAPT mechanism we have identified as *personal significance, insights, and meaningfulness*, given that emotions inform about things that are personally significant and meaningful. The SAPT mechanism of *reassessment of memories*, could also be considered a subset of the experiencing construct, given the memories that are often referenced as being *reassessed* therapeutically in SAPT tend to be emotionally salient and development significant memories. These overlaps have lead for some to argue that substances such as MDMA which functions to enhance the sense of connection, meaningfulness and processing of emotions, make it a natural adjunct for experiential therapies such as emotion focused couples therapy (Almond and Allan, 2019). There, *awe and peak-experiences* potentially amplify emotional signals such as compassion to everything as well as fear.

Whilst the other therapeutic modalities seemed to be technically suited to the integration stage of SAPTs; the humanistic-experiential techniques most closely relate to those that have been described for the administration stage. For example, the current treatment-model for MDMA-assisted psychotherapy for PTSD (Mithoefer, 2017) and some trials for depression and anxiety (Carhart-Harris et al., 2016; Griffiths et al., 2016; Roseman et al., 2018; Palhano-Fontes et al., 2019) suggest a framework-free and predominantly non-directive and client-centered approach seems especially relevant for SAPTs, given the substances function to elicit relevant material. Therefore a process following stance, as is typical in humanistic-experiential therapies, seems the appropriate for this stage of therapy, and that content directive therapeutic techniques could interfere the substances eliciting function. The non-directive approach of MDMA-assisted psychotherapy can empower clients to understand and resolve problems in their lives on their own. This may be reflected in enhanced measures of personal strength and sense of possibility (Gorman et al., 2020) and qualitative reports suggesting that clients prefer non-directive approaches (Watts et al., 2017).

### Synergistic Therapeutic Mechanisms

Nielson and Guss (2018) outline similarities between psychedelic therapy, mindfulness-based interventions, and psychoanalysis. First, they all evoke altered states of consciousness allowing inaccessible parts of the self to be retrieved. As mentioned above, increased cognitive flexibility (i.e., increased bottom-up prediction errors) induced by psychedelics may result in de-patterning and perspective-shifts, increased insights, and personal meaningful re-experiencing of memories. Second, the therapist gives shape and direction to the course of the therapy, but has limited control over the tools for healing which are inherited in the client’s mind (Freud, 1912; Walsh and Grob, 2006; McCown et al., 2010). Third, the therapist’s personal perception, presence, and experience during the therapeutic sessions is used to guide the client and the process (Rubin, 1985; Kabat-Zinn, 2003; Phelps, 2017). In turns, the creation of the ideal set and setting is supported by the therapist, to facilitate an altered state of consciousness that supports the unfolding of the inner healing capacities of the client (Nielson and Guss, 2018).

### Moving Towards an Incorporation of Various Frameworks

In general, psychotherapy usually produces similar outcomes across different orientations and it might be more effective to fit and adapt the intervention to the particular client’s needs (APA, 2012). Likewise, the extensive review of the literature suggests that the effectiveness or reported benefits of SAPT might not be due to the theoretical orientation of the therapist *per se*, but rather derived from important similarities and overlap between different schools of psychotherapy and dependent on intra- and interpersonal variables (Frank and Frank, 1991; Asay and Lambert, 1999; Wampold, 2015). Altered states of consciousness often allow clients to employ state alteration (Jiang et al., 2017) to change their point of view on an issue (as in cognitive restructuring with hypnosis; Spiegel, 2013) or experience negative events and let feelings pass without struggling against them (as in mindfulness; Paulson et al., 2013). Further, memory reactivation, reconsolidation, and reintegration (Lane et al., 2015) during SAPT may aid therapeutic change. This can be aided by CBT, interpersonal psychotherapy, psychodynamic psychotherapy, emotion-focused-therapy, or hypnosis and mindfulness (Lane et al., 2015; Spiegel, 2016). Providing a framework in which the client’s problems can be understood may be more important than a particular orientation (Ludwig and Levine, 1965a).

## Conclusion

In this narrative review, we have outlined valuable prerequisites for thepsists who are considering engaging in SAPT that comprise a preset of knowledge and considerations for investigations and interventions. In doing so, several approaches to SAPT have been outlined to illustrate how literature on various schools of psychotherapy can be linked to the literature we reviewed about SAPT. Thereby, similarities across models and therapeutic orientations were highlighted. From this review, it may be suggested that various schools of psychotherapy could be incorporated into the development of respective therapeutic models. We hope this provides a basis for the development and adaptation of future investigations, therapeutic models, training programs for therapists, and those interested in the therapeutic potential of SAPT.

There are limitations to the findings outlined above since the mechanisms of action and therapeutic models depicted in this article have yet to be validated and further investigation employing rigorous research is required. Moreover, comorbidity is a common issue with substance use, anxiety, mood disorders, and post-traumatic stress (Rytwinski et al., 2013; Lai et al., 2015) – the very conditions for which current clinical investigations of SAPT are being conducted. It may be argued that comorbidity between disorders could facilitate the therapeutic application of SAPT across disorders (Breeksema et al., 2020). Palhano-Fontes et al. (2019), for instance, reported that five participants with TRD and comorbid borderline personality disorder who received Ayahuasca showed significant decreases in clinical symptomps of depression 7 days post treatment. However, treatment of comorbid disoders introduces unique challenges in terms of diagnoses and screening (e.g., Shivani et al., 2002; Wu and Fang, 2014), effective treatments (e.g., Roberts et al., 2015), and potential side-effects (e.g., with bipolar- and/or psychotic disoders). More research regarding the potential harms and benefits of SAPT for the treatment for comorbid disorders and potential transdiagnostic approaches (Dalgleish et al., 2020) is needed. Another limitation pertains to TRD: many forms of TRD belong to the bipolar spectrum and some have argued that treatment-resistant unipolar depression may be considered a prodromal phase of bipolar disorder (Dudek et al., 2010). Non-diagnosed bipolarity was further identified as an independent risk factor for treatment resistance in TRD (Bennabi et al., 2015). The diagnostic distinction between unipolar depression and bipolar depression is of utmost importance because antidepressant medication, commonly used for the treatment of unipolar depression, may induce mania, hypomania, insomnia, and irritability in those with bipolar depression (Ghaemi et al., 2000; Pacchiarotti et al., 2013). Since some people with bipolar diosder may experience psychotic episodes after substance use (Starzer et al., 2018), it is listed as a contraindication for most clinical investiations applying SAPT. However, the safety and efficacy of psilocybin is currently investigated for those with biploar disorder type 2.^1^ For the treatment of TRD with SAPT, the identification of unipolar and bipolar depression (especially bipolar depression type 1) has to be closely monitored during the screening process and more research is needed to improve screening for and treatment of bipolar depression with SAPT.

We have outlined the mechanisms of change in SAPT that have been identified in the literature and their similarity to mechanisms of change described in traditional psychotherapy. Through this analysis it was highlighted that different traditional therapeutic approaches and techniques might be effectively combined with and related to certain stages of SAPT and different therapeutic outcomes. The purposeful application and investigation of different approaches and techniques and their relation to therapeutic outcomes should be assessed in the future.

Furthermore, current literature may suggest that like traditional psychotherapy, the therapeutic effect of SAPT may not only be mediated by specific substances, therapeutic orientations, or models *per se*; rather the therapeutic effect may depend on factors like the therapeutic- and interpersonal relationship, empathy, therapeutic presence, and unconditional acceptance (Asay and Lambert, 1999; Geller and Greenberg, 2012; Wampold, 2015). Despite the validation of current standardized models, future research should investigate these intersecting variables across models, as well. Careful attention to pre-screening, contextual factors (i.e., set and setting), substance and dosage, as well as preparation (intentions) and integration (meaning) of the experience are commonalities across different models of SAPT (Bogenschutz and Ross, 2016) and thought to be key determinants of the therapeutic outcome (Bogenschutz and Forcehimes, 2017). Combining these key determinants with common factors that are important for producing positive treatment outcomes in psychotherapy such as therapeutic relationship, empathy, expectations, and therapist differences (Wampold, 2015), may be important to allow for the broader application of SAPT across different models of psychotherapy (that share these common factors). Consequently, future studies should, as a standard, include valid measures of the therapeutic relationship (Horvath and Greenberg, 1989), therapeutic presence (Geller et al., 2010), empathy (Dziobek et al., 2008), expectations, and personality factors of therapist and client (e.g., Benjamin et al., 2006) in order to assess their potential influence on the treatment outcome. Eventually, it is conceivable that certain substances (or their combination) will be more compatible with certain therapeutic approaches and/or clinical targets, synergistically fostering particular mechanisms. Thus far, no controlled studies have been conducted in which different models of psychotherapy have been compared using the same compound, or in which different substances have been compared using the same models of psychotherapy. More research is needed to determine which substances and forms of therapy may be effectively combined.

## Author Contributions

ST and TW conceived the presented idea, and reviewed and analyzed the literature. The first draft of the manuscript was written by ST, SB, and JS. PS contributed substantial ideas and feedback. All authors discussed the results. All authors contributed to the article and approved the submitted version.

### Conflict of Interest

Stephen Bright is a Director of the Australian not-for-profit company Psychedelic Research in Science & Medicine Pty Ltd (PRISM). PRISM’s mission is to initiate, fund and facilitate psychedelic science in Australia. Stephen Bright has received funding from PRISM to assist with his research, including this paper.

The remaining authors declare that the research was conducted in the absence of any commercial or financial relationships that could be construed as a potential conflict of interest.

## Footnotes

^1^https://clinicaltrials.gov/ct2/show/NCT04433845

## References

[ref5] Adamson S. Metzner R. (1988). The nature of the MDMA experience and its role in healing, psychotherapy and spiritual practice. ReVision 10, 59–72.

[ref6] Agin-Liebes G. I. Malone T. Matthew M. Y. Sarah E. M. Linnae Ponté K. Guss J.. (2020). Long-term follow-up of psilocybin-assisted psychotherapy for psychiatric and existential distress in patients with life-threatening cancer. J. Psychopharmacol. 34, 155–166. 10.1177/0269881119897615 31916890

[ref7] Almond K. Allan R. (2019). Incorporating MDMA as an adjunct in emotionally focused couples therapy with clients impacted by trauma or PTSD. Family J. 27, 293–299. 10.1177/1066480719852360

[ref8] Alper K. R. (2001). Chapter 1 Ibogaine: a review. Alkaloids: Chem. Biol. 59, 1–38. 10.1016/S0099-9598(01)56005-8 11705103

[ref9] Andritzky W. (1989). Sociopsychotherapeutic functions of Ayahuasca healing in Amazonia. J. Psychoactive Drugs 21, 77–89. 10.1080/02791072.1989.10472145 2656954

[ref10] APA (2012). Recognition of psychotherapy effectiveness. Available at: https://www.apa.org/about/policy/resolution-psychotherapy (Accessed July 5, 2020).

[ref11] Asay T. P. Lambert M. J. (1999). “The empirical case for the common factors in therapy: quantitative findings,” in The Heart and Soul of Change: What Works in Therapy. eds. Hubble M. A. Duncan B. L. Miller S. D. (Washington, DC: American Psychological Association), 23–55.

[ref126] Bai Y. Maruskin L. A. Chen S. Gordon A. M. Stellar J. E. McNeil G. D.. (2017). Awe, the diminished self, and collective engagement: universals and cultural variations in the small self. J. Pers. Soc. Psychol. 113, 185–209. 10.1037/pspa0000087 28481617

[ref12] Barnett B. S. Siu W. O. Pope H. G. (2018). A survey of American psychiatrists’ attitudes toward classic hallucinogens. J. Nerv. Ment. Dis. 206, 476–480. 10.1097/NMD.0000000000000828 29781894

[ref13] Barrett F. S. Johnson M. W. Griffiths R. R. (2015). Validation of the revised mystical experience questionnaire in experimental sessions with psilocybin. J. Psychopharmacol. 29, 1182–1190. 10.1177/0269881115609019 26442957 PMC5203697

[ref301] Beck A. T. (1976). Cognitive Therapy and the Emotional Disorders. New York: Meridian.

[ref14] Belouin S. J. Henningfield J. E. (2018). Psychedelics: where we are now, why we got here, what we must do. Neuropharmacology 142, 7–19. 10.1016/j.neuropharm.2018.02.018 29476779

[ref15] Belser A. B. Gabrielle A.-L. Cody Swift T. Terrana S. Devenot N. Friedman H. L.. (2017). Patient experiences of psilocybin-assisted psychotherapy: an interpretative phenomenological analysis. J. Humanist. Psychol. 57, 354–388. 10.1177/0022167817706884

[ref16] Benjamin L. S. Rothweiler J. C. Critchfield K. L. (2006). The use of Structural Analysis of Social Behavior (SASB) as an assessment tool. Annu. Rev. Clin. Psychol. 2, 83–109. 10.1146/annurev.clinpsy.2.022305.095337, PMID: 17716065

[ref17] Bennabi D. Aouizerate B. El-Hage W. Doumy O. Moliere F. Courtet P.. (2015). Risk factors for treatment resistance in unipolar depression: a systematic review. J. Affect. Disord. 171, 137–141. 10.1016/j.jad.2014.09.020, PMID: 25305428

[ref18] Bogenschutz M. P. (2013). Studying the effects of classic hallucinogens in the treatment of alcoholism: rationale, methodology, and current research with psilocybin. Curr. Drug Abuse Rev. 6, 17–29. 10.2174/15733998113099990002, PMID: 23627783

[ref19] Bogenschutz M. P. Samantha K. P. Jessie H. D. Sean S. A. Tara C. M. Lindsey T. O.. (2018). Clinical interpretations of patient experience in a trial of psilocybin-assisted psychotherapy for alcohol use disorder. Front. Pharmacol. 9:100. 10.3389/fphar.2018.00100, PMID: 29515439 PMC5826237

[ref20] Bogenschutz M. P. Forcehimes A. A. (2017). Development of a psychotherapeutic model for psilocybin-assisted treatment of alcoholism. J. Humanist. Psychol. 57, 389–414. 10.1177/0022167816673493

[ref21] Bogenschutz M. P. Forcehimes A. A. Pommy J. A. Wilcox C. E. Barbosa P. C. R. Strassman R. J. (2015). Psilocybin-assisted treatment for alcohol dependence: a proof-of-concept study. J. Psychopharmacol. 29, 289–299. 10.1177/0269881114565144, PMID: 25586396

[ref22] Bogenschutz M. P. Ross S. (2016). “Therapeutic applications of classic hallucinogens,” in Behavioral Neurobiology of Psychedelic Drugs. Current Topics in Behavioral Neurosciences. *Vol*. 36. eds. Halberstadt A. L. Vollenweider F. X. Nichols D. E. (Berlin, Heidelberg: Springer). 10.1007/7854_2016_464 28512684

[ref23] Bouso J. C. dos Santos R. G. Alcázar-Córcoles M. Á. Hallak J. E. C. (2018). Serotonergic psychedelics and personality: a systematic review of contemporary research. Neurosci. Biobehav. Rev. 87, 118–132. 10.1016/j.neubiorev.2018.02.004, PMID: 29452127

[ref24] Breeksema J. J. Niemeijer A. R. Krediet E. Vermetten E. Schoevers R. A. (2020). Psychedelic treatments for psychiatric disorders: a systematic review and thematic synthesis of patient experiences in qualitative studies. CNS Drugs. Adis. 34, 925–946. 10.1007/s40263-020-00748-y, PMID: 32803732 PMC7447679

[ref25] Carbonaro T. M. Bradstreet M. P. Barrett F. S. MacLean K. A. Jesse R. Johnson M. W.. (2016). Survey study of challenging experiences after ingesting psilocybin mushrooms: acute and enduring positive and negative consequences. J. Psychopharmacol. 30, 1268–1278. 10.1177/0269881116662634, PMID: 27578767 PMC5551678

[ref31] Carhart-Harris R. L. Bolstridge M. Rucker J. Day C. M. J. Erritzoe D. Kaelen M.. (2016). Psilocybin with psychological support for treatment-resistant depression: an open-label feasibility study. Lancet Psychiatry 3, 619–627. 10.1016/S2215-0366(16)30065-7, PMID: 27210031

[ref26] Carhart-Harris R. L. Erritzoe D. Haijen E. Kaelen M. Watts R. (2018). Psychedelics and connectedness. Psychopharmacology 235, 547–550. 10.1007/s00213-017-4701-y, PMID: 28795211

[ref27] Carhart-Harris R. L. Friston K. J. (2019). REBUS and the anarchic brain: toward a unified model of the brain action of psychedelics. Pharmacol. Rev. 71, 316–344. 10.1124/pr.118.017160, PMID: 31221820 PMC6588209

[ref28] Carhart-Harris R. L. Kaelen M. Bolstridge M. Williams T. M. Williams L. T. Underwood R.. (2016). The paradoxical psychological effects of lysergic acid diethylamide (LSD). Psychol. Med. 46, 1379–1390. 10.1017/S0033291715002901, PMID: 26847689

[ref29] Carhart-Harris R. L. Kaelen M. Whalley M. G. Bolstridge M. Feilding A. Nutt D. J. (2015). LSD enhances suggestibility in healthy volunteers. Psychopharmacology 232, 785–794. 10.1007/s00213-014-3714-z, PMID: 25242255

[ref32] Carhart-Harris R. L. Leech R. Hellyer P. J. Shanahan M. Feilding A. Tagliazucchi E.. (2014). The entropic brain: a theory of conscious states informed by neuroimaging research with psychedelic drugs. Front. Human Neurosci. 8:20. 10.3389/fnhum.2014.00020, PMID: 24550805 PMC3909994

[ref35] Carhart-Harris R. Nutt D. (2014). Was it a vision or a waking dream? Front. Psychol. 5:255. 10.3389/fpsyg.2014.00255, PMID: 24772095 PMC3983501

[ref30] Carhart-Harris R. L. Nutt D. J. (2017). Serotonin and brain function: a tale of two receptors. J. Psychopharmacol. 31, 1091–1120. 10.1177/0269881117725915, PMID: 28858536 PMC5606297

[ref33] Carhart-Harris R. L. Roseman L. Bolstridge M. Lysia D. Nienke Pannekoek J. Wall M. B.. (2017). Psilocybin for treatment-resistant depression: FMRI-measured brain mechanisms. Sci. Rep. 7:13187. 10.1038/s41598-017-13282-7, PMID: 29030624 PMC5640601

[ref34] Carhart-Harris R. L. Roseman L. Haijen E. Erritzoe D. Watts R. Branchi I.. (2018). Psychedelics and the essential importance of context. J. Psychopharmacol. 32, 725–731. 10.1177/0269881118754710, PMID: 29446697

[ref36] Cattell J. P. (1957). Use of drugs in psychodynamic investigations. Proc. Annu. Meet. Am. Psychopathol. Assoc., 218–235. 13441672

[ref37] Chandler A. L. Hartman M. A. (1960). Lysergic acid diethylamide (LSD-25) as a facilitating agent in psychotherapy. Arch. Gen. Psychiatry 2, 286–299. 10.1001/archpsyc.1960.03590090042008

[ref38] Cohen S. (1960). Lysergic acid diethylamide: side effects and complications. J. Nerv. Ment. Dis. 130, 30–40. 10.1097/00005053-196001000-00005 13811003

[ref39] Costello C. G. (1964). Lysergic acid diethylamide (LSD 25) and behavior therapy. Behav. Res. Ther. 2, 117–129. 10.1016/0005-7967(64)90005-1, PMID: 14212951

[ref40] Cutner M. (1959). Analytic work with LSD 25. Psychiatry Q. 33, 715–757. 10.1007/BF01562041 13813451

[ref41] Dalgleish T. Black M. Johnston D. Bevan A. (2020). Transdiagnostic approaches to mental health problems: current status and future directions. J. Consult. Clin. Psychol. 88, 179–195. 10.1037/ccp0000482, PMID: 32068421 PMC7027356

[ref42] Dudek D. Rybakowski J. K. Siwek M. Pawłowski T. Lojko D. Roczeń R.. (2010). Risk factors of treatment resistance in major depression: association with bipolarity. J. Affect. Disord. 126, 268–271. 10.1016/j.jad.2010.03.001, PMID: 20381154

[ref43] Dziobek I. Rogers K. Fleck S. Bahnemann M. Heekeren H. R. Wolf O. T.. (2008). Dissociation of cognitive and emotional empathy in adults with Asperger syndrome using the Multifaceted Empathy Test (MET). J. Autism Dev. Disord. 38, 464–473. 10.1007/s10803-007-0486-x, PMID: 17990089

[ref44] Eisner B. G. Cohen S. (1958). Psychotherapy with lysergic acid diethylamide. J. Nerv. Ment. Dis. 127, 528–539. 10.1097/00005053-195812000-00006 13621221

[ref302] Ellis A. (1962). Reason and Emotion in Psychotherapy. New York: Lyle Stuart.

[ref45] Elsey J. W. B. (2017). Psychedelic drug use in healthy individuals: a review of benefits, costs, and implications for drug policy. Drug Sci. Policy Law 3:205032451772323. 10.1177/2050324517723232

[ref46] Erritzoe D. Roseman L. Nour M. M. MacLean K. Kaelen M. Nutt D. J.. (2018). Effects of psilocybin therapy on personality structure. Acta Psychiatr. Scand. 138, 368–378. 10.1111/acps.12904, PMID: 29923178 PMC6220878

[ref47] Fadiman J. (2011). The Psychedelic Explorer’s Guide: Safe, Therapeutic, and Sacred Journeys. Rochester, VT: Park Street Press.

[ref48] Fernández X. Santos R. G. D. Cutchet M. Fondevila S. González D. Alcázar M. Á.. (2013). “Assessment of the psychotherapeutic effects of ritual Ayahuasca use on drug dependency: a pilot study,” in The Therapeutic Use of Ayahuasca. eds. Labate B. C. Cavnar C. (Berlin Heidelberg: Springer-Verlag), 183–196.

[ref49] Fischman L. G. (1983). Dreams, hallucinogenic drug states, and schizophrenia: a psychological and biological comparison. Schizophr. Bull. 9, 73–94. 10.1093/schbul/9.1.73, PMID: 6133348

[ref51] Forstmann M. Sagioglou C. (2017). Lifetime experience with (classic) psychedelics predicts pro-environmental behavior through an increase in nature relatedness. J. Psychopharmacol. 31, 975–988. 10.1177/0269881117714049, PMID: 28631526

[ref50] Forstmann M. Sagioglou C. (2021). How psychedelic researchers’ self-admitted substance use and their association with psychedelic culture affect people’s perceptions of their scientific integrity and the quality of their research. Public Underst. Sci. 30, 308–313. 10.1177/0963662520981728 33356921

[ref52] Frank J. D. Frank J. B. (1991). Persuasion and Healing: Comparative Study of Psychotherapy. 3rd *Edn*. Baltimore: Johns Hopkins University Press.

[ref53] Franquesa A. Sainz-Cort A. Gandy S. Soler J. Alcázar-Córcoles M. Á. Bouso J. C. (2018). Psychological variables implied in the therapeutic effect of Ayahuasca: a contextual approach. Psychiatry Res. 264, 334–339. 10.1016/j.psychres.2018.04.012, PMID: 29674223

[ref54] Frecska E. (2011). “The risks and potential benefits of Ayahuasca use from a psychopharmacological perspective,” in The Internationalization of Ayahuasca. eds. Labate B. C. Jungaberle H. (Munster: LIT Verlag), 151–166.

[ref3] Frecska E. Bokor P. Winkelman M. (2016). The therapeutic potentials of Ayahuasca: possible effects against various diseases of civilization. Front. Pharmacol. 7:35. 10.3389/fphar.2016.00035, PMID: 26973523 PMC4773875

[ref55] Freud S. (1912). “Recommendations to physicians practising psycho-analysis,” in The Case of Schreber, Papers on Technique and Other Works Vol. XII (1911–1913). ed. Strachey J. (London: The Hogarth Press and the Institute of Psycho-analysis), 109–120.

[ref56] Freud S. (1920). A General Introduction to Psychoanalysis. North Charleston, South Carolina: Createspace Independent Publishing Platform.

[ref57] Gallimore A. R. (2015). Restructuring consciousness – The psychedelic state in light of integrated information theory. Front. Hum. Neurosci. 9:346. 10.3389/fnhum.2015.00346, PMID: 26124719 PMC4464176

[ref58] Garcia-Romeu A. Richards W. A. (2018). Current perspectives on psychedelic therapy: use of serotonergic hallucinogens in clinical interventions. Int. Rev. Psychiatry 30, 291–316. 10.1080/09540261.2018.1486289, PMID: 30422079

[ref59] Gasser P. (1995). “Die Psycholytische Psychotherapie in Der Schweiz (1988–1993),” in Eine Katamnestische Erhebung. Jahrbuch Für Transkulturelle Medizin und Psychotherapie (Berlin: VWB Verlag für Wissenschaft und Bildung), 143–162.

[ref60] Gasser P. Holstein D. Michel Y. Doblin R. Yazar-Klosinski B. Passie T.. (2014). Safety and efficacy of lysergic acid diethylamide-assisted psychotherapy for anxiety associated with life-threatening diseases. J. Nerv. Ment. Dis. 202, 513–520. 10.1097/NMD.0000000000000113, PMID: 24594678 PMC4086777

[ref61] Gasser P. Kirchner K. Passie T. (2015). LSD-assisted psychotherapy for anxiety associated with a life-threatening disease: a qualitative study of acute and sustained subjective effects. J. Psychopharmacol. 29, 57–68. 10.1177/0269881114555249, PMID: 25389218

[ref62] Geller S. M. Greenberg L. S. (2012). Therapeutic Presence: A Mindful Approach to Effective Therapy. Washington, DC: American Psychological Association.

[ref63] Geller S. M. Greenberg L. S. Watson J. C. (2010). Therapist and client perceptions of therapeutic presence: the development of a measure. Psychother. Res. 20, 599–610. 10.1080/10503307.2010.495957, PMID: 20845229

[ref64] Gendlin E. T. (1996). The Practicings Professional. Focusing-Oriented Psychotherapy: A Manual of the Experiential Method. New York, NY: Guilford Press.

[ref65] Ghaemi S. N. Boiman E. E. Goodwin F. K. (2000). Diagnosing bipolar disorder and the effect of antidepressants: a naturalistic study. J. Clin. Psychiatry 61, 804–808. 10.4088/JCP.v61n1013 11078046

[ref66] Gorman I. Belser A. B. Jerome L. Hennigan C. Shechet B. Hamilton S.. (2020). Posttraumatic growth after MDMA-assisted psychotherapy for posttraumatic stress disorder. J. Trauma. Stress. 33, 161–170. 10.1002/jts.22479, PMID: 32073177 PMC7216948

[ref67] Gowdy J. Krall L. (2015). The economic origins of ultrasociality. Behav. Brain Sci. 39:e92. 10.1017/S0140525X1500059X 25915060

[ref68] Graber C. M. (2010). Eintritt in Heilende Bewusstseinszustände Grundlagen Zur Psycholytischen Praxis. Switzerland: Nachtschatten-Verl.

[ref69] Greenberg J. R. Mitchell S. A. (1983). Object Relations in Psychoanalytic Theory. Cambridge, Massachusetts: Harvard University Press.

[ref70] Greenberg L. S. (2015). Emotion-Focused Therapy: Coaching Clients to Work Through Their Feelings. Washington, DC: American Psychological Association.

[ref71] Greenberg L. S. Johnson S. M. (1988). Emotionally Focused Therapy for Couples. New York, NY: Guilford Press.

[ref72] Greenberg L. S. Rice L. Elliott R. (1993). Process-Experiential Therapy: Facilitating Emotional Change. New York: Guilford Press.

[ref73] Greer G. R. Tolbert R. (1998). A method of conducting therapeutic sessions with MDMA. J. Psychoactive Drugs 30, 371–379. 10.1080/02791072.1998.10399713, PMID: 9924843

[ref74] Grieco A. Bloom R. (1981). Psychotherapy with hallucinogenic adjuncts from a learning perspective. Int. J. Addict. 16, 801–827. 7327766 10.3109/10826088109038891

[ref78] Griffiths R. R. Hurwitz E. S. Davis A. K. Johnson M. W. Jesse R. (2019). Survey of subjective ‘god encounter experiences’: comparisons among naturally occurring experiences and those occasioned by the classic psychedelics psilocybin, LSD, Ayahuasca, or DMT. PLoS One 14:e0214377. 10.1371/journal.pone.0214377, PMID: 31013281 PMC6478303

[ref79] Griffiths R. R. Johnson M. W. Richards W. A. Richards B. D. Jesse R. MacLean K. A.. (2018). Psilocybin-occasioned mystical-type experience in combination with meditation and other spiritual practices produces enduring positive changes in psychological functioning and in trait measures of prosocial attitudes and behaviors. J. Psychopharmacol. 32, 49–69. 10.1177/0269881117731279 29020861 PMC5772431

[ref77] Griffiths R. R. Matthew W. J. Michael A. C. Umbricht A. William A. R. Brian D. R.. (2016). Psilocybin produces substantial and sustained decreases in depression and anxiety in patients with life-threatening cancer: a randomized double-blind trial. J. Psychopharmacol. 30, 1181–1197. 10.1177/0269881116675513, PMID: 27909165 PMC5367557

[ref75] Griffiths R. R. Richards W. A. Johnson M. W. McCann U. D. Jesse R. (2008). Mystical-type experiences occasioned by psilocybin mediate the attribution of personal meaning and spiritual significance 14 months later. J. Psychopharmacol. 22, 621–632. 10.1177/0269881108094300, PMID: 18593735 PMC3050654

[ref76] Griffiths R. R. Richards W. A. McCann U. Jesse R. (2006). Psilocybin can occasion mystical-type experiences having substantial and sustained personal meaning and spiritual significance. Psychopharmacology 187, 268–283. 10.1007/s00213-006-0457-5, PMID: 16826400

[ref80] Grinspoon L. Bakalar J. B. (1979). Psychedelic Drugs Reconsidered. New York: Basic Books.

[ref82] Grof S. (2000). LSD-Psychotherapie. Stuttgart, Germany: Klett-Cotta.

[ref81] Grof S. Goodman L. E. Richards W. A. Kurland A. A. (1973). LSD-assisted psychotherapy in patients with terminal cancer. Int. Pharmacopsychiatry 8, 129–244. 10.1159/000467984 4140164

[ref83] Gucker D. K. (1963). Combining external and internal symbolization in the LSD episode. J. Psychol. 55, 401–408. 10.1080/00223980.1963.9916633 13951059

[ref85] Hartogsohn I. (2016). Set and setting, psychedelics and the placebo response: an extra-pharmacological perspective on psychopharmacology. J. Psychopharmacol. 30, 1259–1267. 10.1177/0269881116677852, PMID: 27852960

[ref84] Hartogsohn I. (2017). Constructing drug effects: a history of set and setting. Drug Sci. Policy Law 3:205032451668332. 10.1177/2050324516683325

[ref86] Hausner M. Dolezal V. (1963). Group and individual psychotherapy under LSD. Adv. Psychosom. Med. 11, 39–59. 10.1159/000285664 14044681

[ref87] Hayes S. C. Strosahl K. Wilson K. G. (1999). Acceptance and Commitment Therapy: The Experiential Approach to Behavior Change. New York, NY: Guilford Press.

[ref88] Hayes S. C. Strosahl K. Wilson K. G. (2012). Acceptance and Commitment Therapy: The Process and Practice of Mindful Change. New York, NY: Guilford Press.

[ref89] Hayes S. C. Wilson K. G. (1994). Acceptance and commitment therapy: altering the verbal support for experiential avoidance. Behav. Anal. 17, 289–303. 10.1007/BF03392677, PMID: 22478193 PMC2733476

[ref91] Hendricks P. S. (2018). Awe: a putative mechanism underlying the effects of classic psychedelic-assisted psychotherapy. Int. Rev. Psychiatry 30, 331–342. 10.1080/09540261.2018.1474185, PMID: 30260256

[ref90] Hendricks P. S. Christopher B. T. Brendan Clark C. David W. C. Matthew W. J. (2015). Classic psychedelic use is associated with reduced psychological distress and suicidality in the United States adult population. J. Psychopharmacol. 29, 280–288. 10.1177/0269881114565653, PMID: 25586402

[ref92] Hofmann A. Ott J. (1980). LSD, My Problem Child. *Vol*. 5 New York: McGraw-Hill.

[ref93] Horvath A. O. Greenberg L. S. (1989). Development and validation of the working alliance inventory. J. Couns. Psychol. 36, 223–233. 10.1037/0022-0167.36.2.223

[ref94] Jacobs B. L. (1978). Dreams and hallucinations: a common neurochemical mechanism mediating their phenomenological similarities. Neurosci. Biobehav. Rev. 2, 59–69. 10.1016/0149-7634(78)90007-6

[ref95] Jesse R. (2001). Code of ethics for spiritual suides. Council on spiritual. 2001. Available at: https://csp.org/docs/code-of-ethics-for-spiritual-guides (Accessed July 5, 2020).

[ref96] Jiang H. White M. P. Greicius M. D. Waelde L. C. Spiegel D. (2017). Brain activity and functional connectivity associated with hypnosis. Cereb. Cortex 27, 4083–4093. 10.1093/cercor/bhw220, PMID: 27469596 PMC6248753

[ref97] Johansen P. O. Krebs T. S. (2015). Psychedelics not linked to mental health problems or suicidal behavior: a population study. J. Psychopharmacol. 29, 270–279. 10.1177/0269881114568039, PMID: 25744618

[ref100] Johnson M. W. Garcia-Romeu A. Griffiths R. R. (2017). Long-term follow-up of psilocybin-facilitated smoking cessation. Am. J. Drug Alcohol Abuse 43, 55–60. 10.3109/00952990.2016.1170135, PMID: 27441452 PMC5641975

[ref99] Johnson M. W. Garcia-Romeu A. Mary P. C. Roland R. G. (2014). Pilot study of the 5-HT _2A_ R agonist psilocybin in the treatment of tobacco addiction. J. Psychopharmacol. 28, 983–992. 10.1177/0269881114548296 25213996 PMC4286320

[ref98] Johnson M. W. Richards W. A. Griffiths R. R. (2008). Human hallucinogen research: guidelines for safety. J. Psychopharmacol. 22, 603–620. 10.1177/0269881108093587, PMID: 18593734 PMC3056407

[ref101] Jungaberle H. Thal S. Zeuch A. Rougemont-Bücking A. von Heyden M. Aicher H.. (2018). Positive psychology in the investigation of psychedelics and entactogens: a critical review. Neuropharmacology 142, 179–199. 10.1016/j.neuropharm.2018.06.034, PMID: 29964094

[ref102] Kabat-Zinn J. (2003). Mindfulness-based interventions in context: past, present, and future. Clin. Psychol. Sci. Pract. 10, 144–156. 10.1093/clipsy.bpg016

[ref103] Kaelen M. Giribaldi B. Raine J. Evans L. Timmerman C. Rodriguez N.. (2018). The hidden therapist: evidence for a central role of music in psychedelic therapy. Psychopharmacology 235, 505–519. 10.1007/s00213-017-4820-5, PMID: 29396616 PMC5893695

[ref104] Kargbo R. B. (2020). Psilocybin therapeutic research: the present and future paradigm. ACS Med. Chem. Lett. 11, 399–402. 10.1021/acsmedchemlett.0c00048, PMID: 32292538 PMC7153026

[ref105] Keltner D. Haidt J. (2003). Approaching awe, a moral, spiritual, and aesthetic emotion. Cogn. Emotion 17, 297–314. 10.1080/02699930302297 29715721

[ref106] Kerr C. E. Josyula K. Littenberg R. (2011). Developing an observing attitude: an analysis of meditation diaries in an MBSR clinical trial. Clin. Psychol. Psychotherapy 18, 80–93. 10.1002/cpp.700, PMID: 21226129 PMC3032385

[ref107] Killingsworth M. A. Gilbert D. T. (2010). A wandering mind is an unhappy mind. Science 330:932. 10.1126/science.1192439, PMID: 21071660

[ref108] Kraehenmann R. (2017). Dreams and psychedelics: neurophenomenological comparison and therapeutic implications. Curr. Neuropharmacol. 15, 1032–1042. 10.2174/1573413713666170619092629, PMID: 28625125 PMC5652011

[ref109] Kraehenmann R. Pokorny D. Vollenweider L. Preller K. H. Pokorny T. Seifritz E.. (2017). Dreamlike effects of LSD on waking imagery in humans depend on serotonin 2A receptor activation. Psychopharmacology 234, 2031–2046. 10.1007/s00213-017-4610-0, PMID: 28386699

[ref110] Krebs T. S. Johansen P. Ø. (2013). Psychedelics and mental health: a population study. PLoS One 8:e63972. 10.1371/journal.pone.0063972, PMID: 23976938 PMC3747247

[ref111] Krupitsky E. M. Grinenko A. Y. (1997). Ketamine psychedelic therapy (KPT): a review of the results of ten years of research. J. Psychoactive Drugs 29, 165–183. 10.1080/02791072.1997.10400185, PMID: 9250944

[ref112] Kuypers K. P. C. Riba J. de la Fuente Revenga M. Barker S. Theunissen E. L. Ramaekers J. G. (2016). Ayahuasca enhances creative divergent thinking while decreasing conventional convergent thinking. Psychopharmacology 233, 3395–3403. 10.1007/s00213-016-4377-8, PMID: 27435062 PMC4989012

[ref1] Lai H. M. X. Cleary M. Sitharthan T. Hunt G. E. (2015). Prevalence of comorbid substance use, anxiety and mood disorders in epidemiological surveys, 1990–2014: a systematic review and meta-analysis. Drug Alcohol Depend. 154, 1–13. 10.1016/j.drugalcdep.2015.05.031, PMID: 26072219

[ref113] Lane R. D. Lee R. Nadel L. Greenberg L. (2015). Memory reconsolidation, emotional arousal, and the process of change in psychotherapy: new insights from brain science. Behav. Brain Sci. 38, 1–64. 10.1017/S0140525X14000041 24827452

[ref114] Leary T. (1961). “Drugs, set & suggestibility.” in Paper Presented at the Annual Meeting of the American Psychological Association. September 6, 1961.

[ref115] Leuner H. (1967). “Present state of psycholytic therapy and its possibilities,” in The Use of LSD in Psychotherapy and Alcoholism. ed. Abramson H. A. (Indianapolis: Bobbs-Merrill), 101–116.

[ref116] Levin R. Nielsen T. (2009). Nightmares, bad dreams, and emotion dysregulation. Curr. Dir. Psychol. Sci. 18, 84–88. 10.1111/j.1467-8721.2009.01614.x

[ref117] Liechti M. E. Dolder P. C. Schmid Y. (2017). Alterations of consciousness and mystical-type experiences after acute LSD in humans. Psychopharmacology 234, 1499–1510. 10.1007/s00213-016-4453-0, PMID: 27714429 PMC5420386

[ref118] Ludwig A. M. Levine J. (1965a). A controlled comparison of five brief treatment techniques employing LSD, hypnosis, and psychotherapy. Am. J. Psychother. 19, 417–435. 10.1176/appi.psychotherapy.1965.19.3.417, PMID: 14339608

[ref119] Ludwig A. M. Levine J. (1965b). Alterations in consciousness produced by combinations of LSD, hypnosis and psychotherapy. Psychopharmacologia 7, 123–137. 10.1097/00005053-196502000-00004 5830970

[ref121] MacLean K. A. Leoutsakos J.-M. S. Johnson M. W. Griffiths R. R. (2012). Factor analysis of the mystical experience questionnaire: a study of experiences occasioned by the hallucinogen psilocybin. J. Sci. Study Relig. 51, 721–737. 10.1111/j.1468-5906.2012.01685.x, PMID: 23316089 PMC3539773

[ref120] MacLean K. A. Matthew W. J. Roland R. G. (2011). Mystical experiences occasioned by the hallucinogen psilocybin lead to increases in the personality domain of openness. J. Psychopharmacol. 25, 1453–1461. 10.1177/0269881111420188, PMID: 21956378 PMC3537171

[ref122] Madsen M. K. Fisher P. M. D. Stenbæk D. S. Kristiansen S. Burmester D. Lehel S.. (2020). A single psilocybin dose is associated with long-term increased mindfulness, preceded by a proportional change in neocortical 5-HT2A receptor binding. Eur. Neuropsychopharmacol. 33, 71–80. 10.1016/j.euroneuro.2020.02.001, PMID: 32146028

[ref123] Majić T. Schmidt T. T. Gallinat J. (2015). Peak experiences and the afterglow phenomenon: when and how do therapeutic effects of hallucinogens depend on psychedelic experiences? J. Psychopharmacol. 29, 241–253. 10.1177/0269881114568040, PMID: 25670401

[ref124] Malone T. C. Mennenga S. E. Guss J. Podrebarac S. K. Owens L. T. Bossis A. P.. (2018). Individual experiences in four cancer patients following psilocybin-assisted psychotherapy. Front. Pharmacol. 9:256. 10.3389/fphar.2018.00256, PMID: 29666578 PMC5891594

[ref125] Mangini M. (1998). Treatment of alcoholism using psychedelic drugs: a review of the program of research. J. Psychoactive Drugs 30, 381–418. 10.1080/02791072.1998.10399714, PMID: 9924844

[ref127] Mason N. L. Mischler E. Uthaug M. V. Kuypers K. P. (2019). Sub-acute effects of psilocybin on empathy, creative thinking, and subjective well-being. J. Psychoactive Drugs 51, 123–134. 10.1080/02791072.2019.1580804, PMID: 30905276

[ref128] Masters R. Houston J. (1966). The Varieties of Psychedelic Experience. New York: Holt, Rinehart & Winston.

[ref129] McCown D. Reibel D. Micozzi M. S. (2010). Teaching mindfulness: a practical guide for clinicians and educators. JAMA 306, 1003–1005. 10.1007/978-0-387-09484-7

[ref130] Mechaneck R. Feldstein S. Dahlberg C. C. Jaffe J. (1968). Experimental investigation of LSD as a psychotherapeutic adjunct. Compr. Psychiatry 9, 490–498. 10.1016/S0010-440X(68)80080-X, PMID: 4879643

[ref131] Metzner R. (1998). Hallucinogenic drugs and plants in psychotherapy and shamanism. J. Psychoactive Drugs 30, 333–341. 10.1080/02791072.1998.10399709, PMID: 9924839

[ref132] Mithoefer M. C. (2017). A manual for MDMA-assisted psychotherapy in the treatment of posttraumatic stress disorder. Available at: https://s3-us-west-1.amazonaws.com/mapscontent/research-archive/mdma/TreatmentManual_MDMAAssistedPsychotherapyVersion+8.1_22+Aug2017.pdf (Accessed June 5, 2020). 10.1002/jts.22696 PMC8453707 34114250

[ref133] Mithoefer M. C. Feduccia A. A. Jerome L. Mithoefer A. Wagner M. Walsh Z.. (2019). MDMA-assisted psychotherapy for treatment of PTSD: study design and rationale for phase 3 trials based on pooled analysis of six phase 2 randomized controlled trials. Psychopharmacology 236, 2735–2745. 10.1007/s00213-019-05249-5, PMID: 31065731 PMC6695343

[ref134] Moreno F. A. Christopher B. W. Keolani Taitano E. Pedro L. D. (2006). Safety, tolerability, and efficacy of psilocybin in 9 patients with obsessive-compulsive disorder. J. Clin. Psychiatry 67, 1735–1740. 10.4088/JCP.v67n1110, PMID: 17196053

[ref135] Morris H. Wallach J. (2014). From PCP to MXE: a comprehensive review of the non-medical use of dissociative drugs. Drug Test. Anal. 6, 614–632. 10.1002/dta.1620, PMID: 24678061

[ref136] Nichols D. E. (2020). Psilocybin: from ancient magic to modern medicine. J. Antibiot. 73, 679–686. 10.1038/s41429-020-0311-8 32398764

[ref137] Nielsen T. Levin R. (2007). Nightmares: a new neurocognitive model. Sleep Med. Rev. 11, 295–310. 10.1016/j.smrv.2007.03.004, PMID: 17498981

[ref138] Nielson E. M. Guss J. (2018). The influence of therapists’ first-hand experience with psychedelics on psychedelic-assisted psychotherapy research and therapist training. J. Psychedelic Stud. 2, 64–73. 10.1556/2054.2018.009

[ref139] Norcross J. C. Lambert M. J. (2018). Psychotherapy relationships that work III. Psychotherapy 55, 303–315. 10.1037/pst0000193, PMID: 30335448

[ref140] Northoff G. (2007). Psychopathology and pathophysiology of the self in depression – neuropsychiatric hypothesis. J. Affect. Disord. 104, 1–14. 10.1016/j.jad.2007.02.012, PMID: 17379318

[ref141] Nour M. M. Evans L. Carhart-Harris R. L. (2017). Psychedelics, personality and political perspectives. J. Psychoactive Drugs 49, 182–191. 10.1080/02791072.2017.1312643, PMID: 28443703

[ref142] Nutt D. J. King L. A. Nichols D. E. (2013). Effects of schedule I drug laws on neuroscience research and treatment innovation. Nat. Rev. Neurosci. 14, 577–585. 10.1038/nrn3530, PMID: 23756634

[ref203] Nuys D. V. (2007). Wise counsel: an interview with Marsha Linehan, Ph.D. on dialectical behavior therapy. Available at: https://podbay.fm/podcast/218827921/e/1192557000 (Accessed July 5, 2020).

[ref143] Pacchiarotti I. Bond D. J. Baldessarini R. J. Nolen W. A. Grunze H. Licht R. W.. (2013). The International Society for Bipolar Disorders (ISBD) task force report on antidepressant use in bipolar disorders. Am. J. Psychiatr. 170, 1249–1262. 10.1176/appi.ajp.2013.13020185, PMID: 24030475 PMC4091043

[ref144] Pahnke W. N. (1969). Psychedelic drugs and mystical experience. Int. Psychiatry Clin. 5, 149–162. PMID: 4892137

[ref145] Pahnke W. N. Kurland A. A. Unger S. Savage C. Grof S. (1971). The experimental use of psychedelic (LSD) psychotherapy. Int. J. Clin. Pharmacol. Ther. Toxicol. 4, 446–454. PMID: 5566727

[ref146] Palhano-Fontes F. Barreto D. Onias H. Andrade K. C. Novaes M. M. Pessoa J. A.. (2019). Rapid antidepressant effects of the psychedelic Ayahuasca in treatment-resistant depression: a randomized placebo-controlled trial. Psychol. Med. 49, 655–663. 10.1017/S0033291718001356, PMID: 29903051 PMC6378413

[ref147] Paulson S. Davidson R. Jha A. Kabat-Zinn J. (2013). Becoming conscious: the science of mindfulness. Ann. N. Y. Acad. Sci. 1303, 87–104. 10.1111/nyas.12203, PMID: 24236866

[ref148] Perls F. Hefferline G. Goodman P. (1951). Gestalt Therapy: Excitement and Growth in the Human Personality. New York: Delta Book.

[ref149] Perls F. S. (1996). Gestalt Therapy Verbatim. Moab, Utha: Real People Press.

[ref150] Phelps J. (2017). Developing guidelines and competencies for the training of psychedelic therapists. J. Humanist. Psychol. 57, 450–487. 10.1177/0022167817711304

[ref151] Pizzagalli D. A. (2011). Frontocingulate dysfunction in depression: toward biomarkers of treatment response. Neuropsychopharmacology 36, 186–206. 10.1038/npp.2010.166 PMC3036952 20861828

[ref152] Pope K. S. (1996). Memory, abuse, and science: questioning claims about the false memory syndrome epidemic. Am. Psychol. 51, 957–974. 10.1037/0003-066X.51.9.957 8819364

[ref153] Preller K. H. Herdener M. Pokorny T. Planzer A. Kraehenmann R. Stämpfli P.. (2017). The fabric of meaning and subjective effects in LSD-induced states depend on serotonin 2A receptor activation. Curr. Biol. 27, 451–457. 10.1016/j.cub.2016.12.030, PMID: 28132813

[ref154] Preller K. H. Razi A. Zeidman P. Stämpfli P. Friston K. J. Vollenweider F. X. (2019). Effective connectivity changes in LSD-induced altered states of consciousness in humans. Proc. Natl. Acad. Sci. U. S. A. 116, 2743–2748. 10.1073/pnas.1815129116, PMID: 30692255 PMC6377471

[ref155] Rhead J. C. Soskin R. A. Turek I. Richards W. A. Yensen R. Kurland A. A.. (1977). Psychedelic drug (DPT)-assisted psychotherapy with alcoholics: a controlled study. J. Psychoactive Drugs 9, 287–300. 10.1080/02791072.1977.10472060

[ref156] Richards W. A. (2017). Psychedelic psychotherapy: insights From 25 years of research. J. Humanist. Psychol. 57, 323–337. 10.1177/0022167816670996

[ref157] Roberts N. P. Roberts P. A. Jones N. Bisson J. I. (2015). Psychological interventions for post-traumatic stress disorder and comorbid substance use disorder: a systematic review and meta-analysis. Clin. Psychol. Rev. 38, 25–38. 10.1016/j.cpr.2015.02.007, PMID: 25792193

[ref158] Rodger J. (2018). Understanding the healing potential of ibogaine through a comparative and interpretive phenomenology of the visionary experience. Anthropol. Conscious. 29, 77–119. 10.1111/anoc.12088

[ref160] Rogers C. R. (1957). The necessary and sufficient conditions of therapeutic personality change. J. Consult. Psychol. 21, 95–103. 10.1037/h0045357, PMID: 13416422

[ref161] Rogers C. R. (1959). A Theory of Therapy, Personality, and Interpersonal Relationships: As Developed in the Client-Centered Framework. New York: McGraw-Hill.

[ref159] Rogers C. R. (1966). Client-Centered Therapy. Washington: American Psychological Association.

[ref162] Rolls A. Makam M. Kroeger D. Colas D. De Lecea L. Craig Heller H. (2013). Sleep to forget: interference of fear memories during sleep. Mol. Psychiatry 18, 1166–1170. 10.1038/mp.2013.121, PMID: 24081009 PMC5036945

[ref4] Roseman L. Nutt D. J. Carhart-Harris R. L. (2018). Quality of acute psychedelic experience predicts therapeutic efficacy of psilocybin for treatment-resistant depression. Front. Pharmacol. 8:974. 10.3389/fphar.2017.00974, PMID: 29387009 PMC5776504

[ref164] Ross S. Bossis A. Guss J. Agin-Liebes G. Malone T. Cohen B.. (2016). Rapid and sustained symptom reduction following psilocybin treatment for anxiety and depression in patients with life-threatening cancer: a randomized controlled trial. J. Psychopharmacol. 30, 1165–1180. 10.1177/0269881116675512, PMID: 27909164 PMC5367551

[ref163] Ross A. Potter G. R. Barratt M. J. Aldridge J. A. (2020). ‘Coming out’: stigma, reflexivity and the drug researcher’s drug use. Contemp. Drug Probl. 47, 268–285. 10.1177/0091450920953635

[ref165] Rubin J. B. (1985). Meditation and psychoanalytic listening. Psychoanal. Rev. 72, 599–613. PMID: 3938031

[ref166] Rudd M. Vohs K. D. Aaker J. (2012). Awe expands People’s perception of time, alters decision making, and enhances well-being. Psychol. Sci. 23, 1130–1136. 10.1177/0956797612438731, PMID: 22886132

[ref167] Rytwinski N. K. Scur M. D. Feeny N. C. Youngstrom E. A. (2013). The co-occurrence of major depressive disorder among individuals with posttraumatic stress disorder: a meta-analysis. J. Trauma. Stress 26, 299–309. 10.1002/jts.21814, PMID: 23696449

[ref168] Sandison R. A. (1959). The role of psychotropic drugs in group therapy. Bull. World Health Organ. 21, 505–515. PMID: 14441417 PMC2537975

[ref169] Sandison R. A. Whitelaw J. D. (1957). Further studies in the therapeutic value of lysergic acid diethylamide in mental illness. J. Ment. Sci. 103, 332–343. 10.1192/bjp.103.431.332, PMID: 13429304

[ref170] Sanz C. Tagliazucchi E. (2018). The experience elicited by hallucinogens presents the highest similarity to dreaming within a large database of psychoactive substance reports. Front. Neurosci. 12:7. 10.3389/fnins.2018.00007, PMID: 29403350 PMC5786560

[ref171] Schenberg E. E. (2018). Psychedelic-assisted psychotherapy: a paradigm shift in psychiatric research and development. Front. Pharmacol. 9:733. 10.3389/fphar.2018.00733, PMID: 30026698 PMC6041963

[ref172] Schiller D. Monfils M. H. Raio C. M. Johnson D. C. Ledoux J. E. Phelps E. A. (2010). Preventing the return of fear in humans using reconsolidation update mechanisms. Nature 463, 449–453. 10.1038/nature08637 PMC3640262 20010606

[ref173] Schmid Y. Liechti M. E. (2018). Long-lasting subjective effects of LSD in normal subjects. Psychopharmacology 235, 535–545. 10.1007/s00213-017-4733-3 28918441 PMC5813062

[ref174] Segal Z. V. Williams J. M. G. Teasdale J. D. (2002). Mindfulness-Based Cognitive Therapy for Depression: A New Approach to Preventing Relapse. New York, NY: Guilford Press.

[ref175] Serafini G. Howland R. H. Rovedi F. Girardi P. Amore M. (2014). The role of ketamine in treatment-resistant depression: a systematic review. Curr. Neuropharmacol. 12, 444–461. 10.2174/1570159X12666140619204251, PMID: 25426012 PMC4243034

[ref177] Sessa B. (2012). The Psychedelic Renaissance: Reassessing the Role of Psychedelic Drugs in 21st Century Psychiatry and Society. London, UK: Muswell Hill Press.

[ref176] Sessa B. (2017). MDMA and PTSD treatment: ‘PTSD: from novel pathophysiology to innovative therapeutics’. Neurosci. Lett. 649, 176–180. 10.1016/j.neulet.2016.07.004, PMID: 27394687

[ref178] Sessa B. Higbed L. Nutt D. (2019). A review of 3,4-Methylenedioxymethamphetamine (MDMA)-assisted psychotherapy. Front. Psych. 10:138. 10.3389/fpsyt.2019.00138, PMID: 30949077 PMC6435835

[ref179] Sessa B. Sakal C. O’Brien S. Nutt D. (2019). First study of safety and tolerability of 3,4-Methylenedioxymethamphetamine (MDMA)-assisted psychotherapy in patients with alcohol use disorder: preliminary data on the first four participants. BMJ Case Rep. 12:e230109. 10.1136/bcr-2019-230109, PMID: 31308191 PMC6663239

[ref180] Shanon B. (2003). Altered states and the study of consciousness — The case of Ayahuasca. J. Mind Behav. 24, 125–153. 10.2307/43853997

[ref181] Shapiro S. L. Carlson L. E. Astin J. A. Freedman B. (2006). Mechanisms of mindfulness. J. Clin. Psychol. 62, 373–386. 10.1002/jclp.20237, PMID: 16385481

[ref182] Sherwood J. N. Stolaroff M. J. Harman W. W. (1968). The psychedelic experience — a new concept in psychotherapy. J. Psychoactive Drugs 1, 96–111. 10.1080/02791072.1968.10524522 13977209

[ref183] Shivani R. Jeffrey Goldsmith R. Anthenelli R. M. (2002). Alcoholism and psychiatric disorders: diagnostic challenges. Alcohol Res. Health 26, 90–98.

[ref184] Sloshower J. Guss J. Krause R. Wallace R. M. Williams M. T. Reed S.. (2020). Psilocybin-assisted therapy of major depressive disorder using acceptance and commitment therapy as a therapeutic frame. J. Contextual Behav. Sci. 15, 12–19. 10.1016/j.jcbs.2019.11.002

[ref185] Smigielski L. Scheidegger M. Kometer M. Franz X. V. (2019). Psilocybin-assisted mindfulness training modulates self-consciousness and brain default mode network connectivity with lasting effects. NeuroImage 196, 207–215. 10.1016/j.neuroimage.2019.04.009, PMID: 30965131

[ref186] Soler J. Elices M. Dominguez-clavé E. Juan C. P. Barrett F. S. (2018). Four weekly Ayahuasca sessions Lead to increases in ‘acceptance’ capacities: a comparison study with a standard 8-week mindfulness training program. Front. Pharmacol. 9:224. 10.3389/fphar.2018.00224, PMID: 29615905 PMC5869920

[ref187] Soler J. Elices M. Franquesa A. Barker S. (2016). Exploring the therapeutic potential of Ayahuasca: acute intake increases mindfulness-related capacities. Psychopharmacology 223, 823–829. 10.1007/s00213-015-4162-0 26612618

[ref188] Spencer A. M. (1964). Modifications in the technique of LSD therapy. Compr. Psychiatry 5, 232–252. 10.1016/S0010-440X(64)80003-1, PMID: 14187116

[ref189] Speth J. Speth C. Kaelen M. Astrid M. S. Feilding A. David J. N.. (2016). Decreased mental time travel to the past correlates with default-mode network disintegration under lysergic acid diethylamide. J. Psychopharmacol. 30, 344–353. 10.1177/0269881116628430, PMID: 26979587

[ref191] Spiegel D. (2013). Tranceformations: hypnosis in brain and body. Depress. Anxiety 30, 340–352. 10.1002/da.22046 23423952

[ref190] Spiegel D. (2016). Psilocybin-assisted psychotherapy for dying cancer patients – aiding the final trip. J. Psychopharmacol. 30, 1215–1217. 10.1177/0269881116675783, PMID: 27909174

[ref2] Starzer M. S. K. Nordentoft M. Hjorthøj C. (2018). Rates and predictors of conversion to schizophrenia or bipolar disorder following substance-induced psychosis. Am. J. Psychiatr. 175, 343–350. 10.1176/appi.ajp.2017.17020223 29179576

[ref192] Stolaroff M. (2004). The Secret Chief Revealed: Conversations With a Pioneer of the Underground Therapy Movement. Florida, FL: MAPS.

[ref193] Strassman R. (2001). DMT: The Spirit Molecule: A Doctor’s Revolutionary Research into the Biology of Near-Death and Mystical Experiences. New York, NY: Simon and Schuster.

[ref194] Swanson L. R. (2018). Unifying theories of psychedelic drug effects. Front. Pharmacol. 9:172. 10.3389/fphar.2018.00172, PMID: 29568270 PMC5853825

[ref195] Swift T. C. Belser A. B. Agin-Liebes G. Devenot N. Terrana S. Friedman H. L.. (2017). Cancer at the dinner table: experiences of psilocybin-assisted psychotherapy for the treatment of cancer-related distress. J. Humanist. Psychol. 57, 488–519. 10.1177/0022167817715966

[ref196] Tagliazucchi E. Roseman L. Kaelen M. Orban C. Muthukumaraswamy S. D. Murphy K.. (2016). Increased global functional connectivity correlates with LSD-induced ego dissolution. Curr. Biol. 26, 1043–1050. 10.1016/j.cub.2016.02.010, PMID: 27085214

[ref197] Thal S. B. Lommen M. J. J. (2018). Current perspective on MDMA-assisted psychotherapy for posttraumatic stress disorder. J. Contemp. Psychother. 48, 99–108. 10.1007/s10879-017-9379-2, PMID: 29720767 PMC5917000

[ref198] Thomas G. Philippe L. Rielle Capler N. Tupper K. W. Martin G. (2013). Ayahuasca-assisted therapy for addiction: results from a preliminary observational study in Canada. Curr. Drug Abuse Rev. 6, 30–42. 10.2174/15733998113099990003, PMID: 23627784

[ref199] Thompson E. (ed) (2014). Waking, Dreaming, Being: Self and Consciousness in Neuroscience, Meditation, and Philosophy. New York: Columbia University Press.

[ref200] Timmermann C. Watts R. Dupuis D. (2020). Towards psychedelic apprenticeship: developing a gentle touch for the mediation and validation of psychedelic-induced insights and revelations. Transcult. Psychiatry. 10.31234/osf.io/j5768 (in press). PMC9660279 35313754

[ref201] Uthaug M. V. van Oorsouw K. Kuypers K. P. C. van Boxtel M. Broers N. J. Mason N. L.. (2018). Sub-acute and long-term effects of Ayahuasca on affect and cognitive thinking style and their association with ego dissolution. Psychopharmacology 235, 2979–2989. 10.1007/s00213-018-4988-3, PMID: 30105399 PMC6182612

[ref202] Valdesolo P. Graham J. (2014). Awe, uncertainty, and agency detection. Psychol. Sci. 25, 170–178. 10.1177/0956797613501884, PMID: 24247728

[ref204] Wagner A. C. Michael C. M. Ann T. M. Candice M. (2019). Combining cognitive-behavioral conjoint therapy for PTSD with 3,4-Methylenedioxymethamphetamine (MDMA): a case example combining cognitive-behavioral conjoint therapy for PTSD with. J. Psychoactive Drugs 51, 166–173. 10.1080/02791072.2019.1589028, PMID: 30890035

[ref205] Walsh R. Grob C. S. (2006). Early psychedelic investigators reflect on the psychological and social implications of their research. J. Humanist. Psychol. 46, 432–448. 10.1177/0022167806286745

[ref206] Walsh Z. Thiessen M. S. (2018). Psychedelics and the new behaviourism: considering the integration of third-wave behaviour therapies with psychedelic-assisted therapy. Int. Rev. Psychiatry 30, 343–349. 10.1080/09540261.2018.1474088, PMID: 30251904

[ref207] Wampold B. E. (2015). How important are the common factors in psychotherapy? An update. World Psychiatry 14, 270–277. 10.1002/wps.20238, PMID: 26407772 PMC4592639

[ref208] Watts R. Day C. Krzanowski J. Nutt D. Carhart-Harris R. (2017). Patients’ accounts of increased ‘connectedness’ and ‘acceptance’ after psilocybin for treatment-resistant depression. J. Humanist. Psychol. 57, 520–564. 10.1177/0022167817709585

[ref209] Watts R. Luoma J. B. (2020). The use of the psychological flexibility model to support psychedelic assisted therapy. J. Contextual Behav. Sci. 15, 92–102. 10.1016/j.jcbs.2019.12.004

[ref210] Wilkins L. K. Girard T. A. Allan Cheyne J. (2011). Ketamine as a primary predictor of out-of-body experiences associated with multiple substance use. Conscious. Cogn. 20, 943–950. 10.1016/j.concog.2011.01.005, PMID: 21324714

[ref211] Wolff M. Evens R. Mertens L. J. Koslowski M. Betzler F. Gründer G.. (2020). Learning to let go: a cognitive-behavioral model of how psychedelic therapy promotes acceptance. Front. Psych. 11:5. 10.3389/fpsyt.2020.00005, PMID: 32153433 PMC7046795

[ref212] Wu Z. Fang Y. (2014). Comorbidity of depressive and anxiety disorders, challenges in diagnosis and assessment. Shanghai Arch. Psychiatry 26, 227–231. 10.3969/j.issn.1002-0829.2014.04.006, PMID: 25317009 PMC4194005

